# Stimuli‐Responsive Nanoparticles for Controlled Drug Delivery in Synergistic Cancer Immunotherapy

**DOI:** 10.1002/advs.202103444

**Published:** 2021-12-19

**Authors:** Jin Zhang, Yandai Lin, Zhe Lin, Qi Wei, Jiaqi Qian, Renjie Ruan, Xiancai Jiang, Linxi Hou, Jibin Song, Jianxun Ding, Huanghao Yang

**Affiliations:** ^1^ Qingyuan Innovation Laboratory College of Chemical Engineering Fuzhou University 2 Xueyuan Road Fuzhou 350108 P. R. China; ^2^ Ruisi (Fujian) Biomedical Engineering Research Center Co Ltd Fuzhou 350100 P. R. China; ^3^ Key Laboratory of Polymer Ecomaterials Changchun Institute of Applied Chemistry Chinese Academy of Sciences 5625 Renmin Street Changchun 130022 P. R. China; ^4^ State Key Laboratory of Molecular Engineering of Polymers Fudan University 220 Handan Road Shanghai 200433 P. R. China; ^5^ MOE Key Laboratory for Analytical Science of Food Safety and Biology State Key Laboratory of Photocatalysis on Energy and Environment College of Chemistry Fuzhou University 2 Xueyuan Road Fuzhou 350108 P. R. China

**Keywords:** clinical translation, controlled drug release, nanotechnology, smart nanocarrier, synergistic immunotherapy

## Abstract

Cancer immunotherapy has achieved promising clinical progress over the recent years for its potential to treat metastatic tumors and inhibit their recurrences effectively. However, low patient response rates and dose‐limiting toxicity remain as major dilemmas for immunotherapy. Stimuli‐responsive nanoparticles (srNPs) combined with immunotherapy offer the possibility to amplify anti‐tumor immune responses, where the weak acidity, high concentration of glutathione, overexpressions of enzymes, and reactive oxygen species, and external stimuli in tumors act as triggers for controlled drug release. This review highlights the design of srNPs based on tumor microenvironment and/or external stimuli to combine with different anti‐tumor drugs, especially the immunoregulatory agents, which eventually realize synergistic immunotherapy of malignant primary or metastatic tumors and acquire a long‐term immune memory to prevent tumor recurrence. The authors hope that this review can provide theoretical guidance for the construction and clinical transformation of smart srNPs for controlled drug delivery in synergistic cancer immunotherapy.

## Graphical abstract

Stimuli‐responsive nanoparticles activated by endo/exo‐microenvironments, including acidity, high‐level glutathione, overexpressed enzymes, and reactive oxygen species, various external stimuli, and so forth, are developed to deliver immunoregulatory agents controllably to eventually amplify the synergistic immunotherapy efficacy of primary or metastatic malignant tumors and acquire a long‐term immune memory to prevent tumor recurrence.

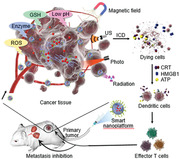

## 1 Introduction

Conventional tumor therapies, such as chemotherapy, radiotherapy (RT), and surgery, have limited therapeutic effects against advanced cancers.^[^ ^1^ ^]^ Owing to the clinical success of immune checkpoint inhibitors, immunotherapy has been established as a crucial pillar of cancer therapy.^[^ ^2^ ^]^ Cancer immunotherapy has the potential to scavenge tumor cells through training the host lymphoid tissue and immune cells in the tumor microenvironment (TME).^[^ ^3^ ^]^ To further enhance immunotherapy efficiency, some in situ vaccination strategies, including photodynamic therapy (PDT), photothermal therapy (PTT), immune agonist therapy, and even chemotherapy, have been employed to induce an effective immune response.^[^ ^4^ ^]^ The activated immune system promotes immune surveillance, eliminates primary, or distant metastatic cancer, establishes immune memory, and regulates immune protection toward tumor relapse.^[^ ^5^ ^]^ Nonetheless, challenges remain to be solved to realize the broad application of cancer immunotherapy in clinic.

One of the hurdles of cancer immunotherapy is the limited response of immune drugs or immune adjuvants. According to clinical research statistics, only a tiny fraction of patients, generally 10–30% depending on cancer type, respond to these immune reagents.^[^ ^6^ ^]^ On the one hand, patients with “cold” tumors (non‐immunogenic tumors) are primarily characterized by insufficient infiltration of immune cells and their low expression level of programmed cell death ligand 1 (PD‐L1) that poorly responds to immune checkpoint inhibitors.^[^ ^7^ ^]^ Another crucial factor is that immune checkpoint inhibitors involve the administration of monoclonal inhibitors in the system, which is prone to induce off‐target side effects via the over‐activation of self‐antigen‐reactive T cells.^[^ ^8^ ^]^ Some patients are treated with immune checkpoint inhibitors or receive immune agonists, while damages of additional complications and infections caused by these uncontrolled immune‐related systemic cytokine storm and cardiotoxicity severely restrict the further application of cancer immunotherapy.^[^ ^9^ ^]^ Hence, new approaches are necessary to amplify the immune response of anti‐tumor T cells by converting “cold tumors” into “hot tumors” and minimizing off‐target toxicity.^[^ ^10^ ^]^

**Scheme 1:**
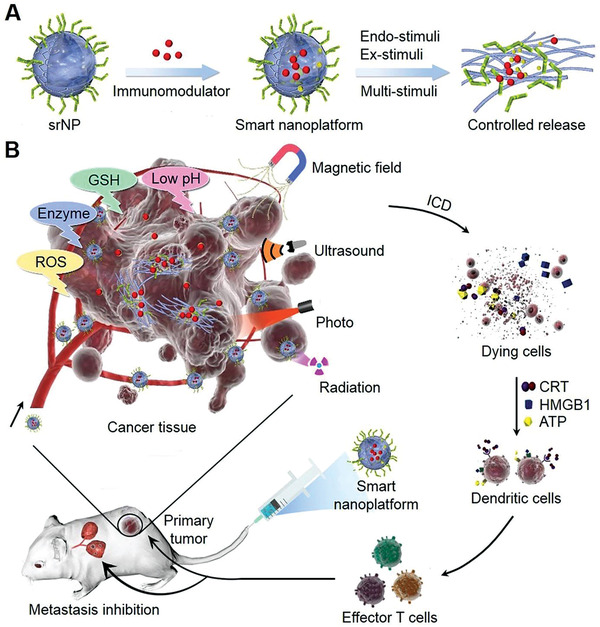
srNPs for controlled drug delivery in synergistic cancer immunotherapy. A) Design of srNPs activated by endo‐ and/or ex‐stimuli, including weak acidity, enzyme, high ROS/GSH concentration, photon, US, magnetic field, and radiation, for smart drug release. (B) srNPs activated by endo‐ and/or ex‐stimuli with controlled immunomodulator release for primary tumor treatment. In addition, dying cells with the expressions of CRT, HMGB1, and ATP “eat me” signals are captured for maturing DC and then are presented to effector T cells (CD8^+^ and CD4^+^ T cells). These activated T cells are then accumulated and attack both the primary and metastatic cancer.

Smart stimuli‐responsive nanoparticles (srNPs) have been widely investigated as efficient drug delivery vehicles in tumor immunotherapy during the last decades.^[^ ^11^ ^]^ Specifically, weak acidity, high concentration of glutathione (GSH), and overexpression of enzymes and reactive oxygen species (ROS) at the tumor site can be utilized as triggers to achieve targeted drug delivery (**Scheme** **1**A). Functionalized srNPs can also be activated by external stimuli including radiation‐, photo‐, ultrasound‐ (US), and magnetic field for realizing PDT, PTT, sono‐dynamic therapy (SDT), and so forth.^[^ ^12^ ^]^ Compared with conventional nanomedicine, the endo‐stimuli‐responsive nanoparticles (en‐srNPs) and exo‐stimuli‐responsive nanoparticles (ex‐srNPs) can increase the immune response rate of patients by regulating TME and turning “cold tumors” into “hot tumors”.^[^ ^13^ ^]^ Specifically, cancer injuries caused by these srNPs may lead to the release of some tumor‐associated antigens (TAAs) in apoptotic and necrotic tumor cells or debris, thus inducing immune response along with the uptake of TAAs by antigen‐presenting cells (APCs).^[^ ^14^ ^]^ Overall, srNPs combined with anti‐tumor immunomodulatory agents can activate intensely synergistic immunotherapy in tumor sites via accurately controlled drug release, offering the possibility to amplify anti‐tumor immune response safely and effectively.^[^ ^15^ ^]^

This review highlights the latest design strategies of srNPs and their combination with different immunoregulatory agents, which exert synergetic therapeutic effects and prevent tumor recurrence efficiently. As shown in Scheme 1B, srNPs activated by locally internal and/or external stimuli are established for preliminary tumor treatment and intelligent drug delivery. Various combinations of the srNPs with loaded chemical drugs and/or immune drugs are listed in **Table** **1**. These srNPs can eliminate cascade malignant tumors synergistically and establish long‐term immune memory by assembling different functions. In detail, calprotectin (CRT), high mobility group box 1 (HMGB1), and adenosine triphosphate (ATP) exposed by apoptotic and necrotic tumor cells as well as their debris activate matured dendritic cells (DCs) and are presented to CD8^+^ and CD4^+^ T cells.^[^ ^64^ ^]^ Then, secretion cytokines (IL‐12 and TNF‐α) from these activated T cells are accumulated at the tumor site and thus attack both the primary and metastatic cancers. In the meantime, the release of chemotherapeutic drugs (e.g., doxorubicin (DOX), paclitaxel (PTX), camptothecin (CPT)), and immune checkpoint antibodies (e.g., anti‐CTLA‐4 anti‐PD‐1, anti‐PD‐L1 inhibitors), immune adjuvants, and enzymes further enhance anti‐tumor efficiency from aspects of cascade immune activator and chemotherapy.^[^ ^65^ ^]^ Given the advantages of cancer‐specific accumulation, deep penetration, and high drug loading efficacy, srNPs are potent for improved synergistic immunotherapy.

**Table 1:** srNPs activated by internal and/or external stimuli for smart drug release and synergistic cancer immunotherapy

Stimulus	Responsive moiety	Immunomodulator	Tumor type	Ref.
pH	pH‐cleavage: Amide bond pH‐sponge: PDEA	PD‐L1 blockade siRNA and photosensitizer	Melanoma	^[^ ^16^ ^]^
	pH‐cleavage: Amide bond	Isothiocyanate, MEK‐, BRAF‐inhibitor, and anti‐PD‐1	Metastatic melanoma	^[^ ^17^ ^]^
	pH‐protonation: PAA	Au and DOX	Hepatoma	^[^ ^18^ ^]^
	pH‐swelling: Tetraethylenepentamine	RNA interference	Prostatic carcinoma	^[^ ^19^ ^]^
	pH‐responsive size shrinkage: Di‐*tert*‐butyl dicarbonate	DOX	Breast carcinoma	^[^ ^20^ ^]^
	pH‐responsive shell‐cleavage: Boronate ester bond	Bortezomib	Breast carcinoma	^[^ ^21^ ^]^
GSH	GSH‐cleavage: Disulfide‐bridge	Photosensitizer Ce6, anti‐CTLA‐4 antibody, and Fe_3_O_4_	Breast carcinoma	^[^ ^22^ ^]^
	GSH‐cleavage: Tetrasulfide bond	DOX and PD‐L1 antibody	Breast carcinoma	^[^ ^23^ ^]^
	GSH‐cleavage: Disulfide‐bond	Photosensitizer and IDO‐1	Breast carcinoma and colorectal cancer	^[^ ^24^ ^]^
	GSH‐cleavage: Disulfide‐bond	Photosensitizer and IDO inhibitor	Breast carcinoma	^[^ ^25^ ^]^
	Redox‐sensitive linkage: Diselenide bond	PTX	Breast carcinoma	^[^ ^26^ ^]^
	GSH‐cleavage: Disulfide‐bond	Docetaxel and Chondroitin sulfate	Breast carcinoma	^[^ ^27^ ^]^
Enzyme	β‐glucuronidase‐degradation: Glucuronide	Imidazoquinoline TLR7/8 agonist	Raw blue reporter cell line	^[^ ^28^ ^]^
	HAase‐responsive: HA	Cilengitide and TNF‐related apoptosis inducing ligand	Human breast carcinoma	^[^ ^29^ ^]^
	Enzyme‐degradation: Matrix metalloproteinases peptide	TLR7 agonist 1V209 and Cy 5.5 dye	Breast carcinoma	^[^ ^30^ ^]^
	Enzyme responsive: Gly‐phe‐leu‐gly tetrapeptide linker	DOX	Breast carcinoma	^[^ ^31^ ^]^
	Enzyme‐degradation: Matrix metalloproteinases peptide	PD‐L1 inhibitor and DOX	Melanoma	^[^ ^32^ ^]^
	Enzyme‐responsive: Polytyrosine	DOX	Colorectal carcinoma	^[^ ^33^ ^]^
ROS	H_2_O_2_‐reaction: MnO_2_	Gold‐photosensitizer and MnO_2_	Breast carcinoma	^[^ ^34^ ^]^
	ROS‐trigger: Tellurium nanowire	Bovine serum albumin and dextran	Breast carcinoma	^[^ ^35^ ^]^
	H_2_O_2_‐reaction: MnO_2_	Glycolysis inhibitor and lactate oxidase	Melanoma	^[^ ^36^ ^]^
	H_2_O_2_‐reaction: MnO_2_	Acriflavine	Breast carcinoma	^[^ ^37^ ^]^
	ROS‐cleavage: Thioketal bond	CPT, photosensitizer, and Pt	Colon cancer	^[^ ^38^ ^]^
	ROS‐responsive: Thioether	PTX and Ce6	Breast carcinoma	^[^ ^39^ ^]^
Photo	Photo‐responsive: Photosensitizer PpIX	PpIX and immune checkpoint inhibitor 1MT	Colorectal carcinoma	^[^ ^40^ ^]^
	Photo‐cleavage: O‐nitrobenzyl	DOX	Cervical carcinoma	^[^ ^41^ ^]^
	Photo‐degradation: 9,10‐dialkoxy‐anthracene based precursor	Graphene quantum dot	Breast carcinoma	^[^ ^42^ ^]^
	Photothermal decomposition: PPP	Fe/FeO nanocrystal, ICG, and DOX	Oral epithelial carcinoma	^[^ ^43^ ^]^
	Photo‐responsive: Chloride disulfonic acid	CPT and photosensitizer Al(III) phthalocyanine chloride disulfonic acid	Breast carcinoma	^[^ ^44^ ^]^
US	US‐responsive: Methoxyethyl methacrylate	DOX	Cervical carcinoma	^[^ ^45^ ^]^
	US‐responsive: US contrast agent microbubble	Interfering RNA	Cervical carcinoma	^[^ ^46^ ^]^
	US‐triggered: Ce6 ester	DOX and Ce6	Fibrosarcoma	^[^ ^47^ ^]^
	US‐responsive: Erythrocyte membrane	DOX and hermimether	Hepatoma carcinoma	^[^ ^48^ ^]^
	US‐responsive: 2‐tetrahydropyranyl methacrylate	DOX	Prostatic cancer	^[^ ^49^ ^]^
Magnetic field	Magnetic ferrite	Fe_3_O_4_ and MnFe_2_O_4_	Breast carcinoma	^[^ ^50^ ^]^
	Mn–Zn ferrite	Mn‐Zn ferrite	Breast carcinoma	^[^ ^51^ ^]^
	Domain iron oxide	Ferrimagnetic vortex and PD‐L1 blockade	Breast carcinoma	^[^ ^52^ ^]^
	Magnetization and superparamagnetism	PD‐1 antibody	Breast carcinoma	^[^ ^53^ ^]^
	Superparamagnetic iron oxide	Fe_3_O_4_ and L‐arginine	Breast carcinoma	^[^ ^54^ ^]^
Radiation	Radiation‐cleavage: Diselenide bond	DOX and PD‐L1 checkpoint blockade	Breast carcinoma	^[^ ^55^ ^]^
	Radiation splinter: Au nanocarrier	aPD‐L1	Colorectal carcinoma	^[^ ^56^ ^]^
	Radiation‐diffusion: Adenine‐modified ZnS	DOX	Glioma	^[^ ^57^ ^]^
	Radiation‐responsive mesoporous silica shells of Eu^3+^‐doped NaGdF_4_	ICG and s‐nitrosothiol group	Breast carcinoma	^[^ ^58^ ^]^
	Radiation‐responsive splintery snowflake‐like Au	aPD‐L1, Au, and Ag nanocrystal	Pancreatic carcinoma	^[^ ^56^ ^]^
Photo/hypoxia	Light‐triggered/hypoxia‐responsive: 2‐nitroimidazole‐grafted conjugated polymer	DOX	Cervical carcinoma	^[^ ^59^ ^]^
Photo/pH	pH/light responsive: Poly(N‐isopropylacrylamide‐*co*‐acrylic acid)	DOX and photosensitizer	Cervical carcinoma	^[^ ^60^ ^]^
pH/thermal	pH‐responsive: Acrylamide; thermal‐responsive: Poly(acrylamide‐*co*‐acrylonitrile)‐PEG	Bovine lactoferricin and HA	Melanoma	^[^ ^61^ ^]^
Photo/pH/hyperthermia	pH‐cleavage: Amide groups; Photo‐cleavage: Ester bond; Photo hyperthermia: Cypate	Cypate and DOX	Breast carcinoma	^[^ ^62^ ^]^
pH/GSH/enzyme	PEG‐peptide‐poly(*ω*‐pentadecalacto‐ne‐*co*‐*N*‐methyldiethyleneamine‐*co*‐thiodipropionate) block	Ce6 and sorafenib	Lung carcinoma	^[^ ^63^ ^]^

Abbreviations: aPD‐L1, anti‐programmed death ligand 1; Ce6, chlorin e6; HA, hyaluronic acid; ICG, indocyanine green; IDO‐1, indoleamine 2,3‐dioxygenase 1; PAA, polyacrylic acid; PDEA, poly(2‐(diethylamino) ethyl methacrylate; PPP, PLGA−polyethylene glycol−poly(*N*‐isopropyl acrylamide); TNF, tumor necrosis factor.

## 2 Endo‐Stimuli‐Responsive Nanoparticles (en‐srNPs) for Controlled Drug Release and Synergistic Cancer Immunotherapy

### 2.1 pH‐Mediated en‐srNPs

The poor uptake and incomplete drug release in cancer cells are the two crucial challenges hampering the clinical applications of nanomedicine.^[^ ^66^ ^]^ Typically, TME is more acidic and always has a lower pH value compared to that of normal tissue, which is primarily credited to the creation of lactic acid in the anaerobic microenvironment and the formation of protons hydrolyzed during the process of ATP.^[^ ^67^ ^]^ To enhance the therapeutic effect of nanoparticles, a tremendous amount of research has been devoted to fabricating TME‐responsive or cancer‐targeted drug delivery platforms.^[^ ^68^ ^]^ The prodrugs composed of therapeutic drugs and hydrophilic polymers via pH‐sensitive linkers of disulfide or/and hydrazone bonds are prone to assemble into amphiphilic srNPs and achieve observable advantages of high drug loading, preferred stability in normal tissue, and controlled drug delivery at tumor sites.^[^ ^69^ ^]^ Consequently, the design of pH‐responsive en‐srNPs has been motivated for inducing intelligent drug release and synergistic cancer immunotherapy.^[^ ^70^ ^]^

The breakage of acid‐sensitive chemical bonds, protonation of tertiary amine groups, and ionization of weak acids are generally the crucial factors that control drug delivery. Cai et al. developed a smart drug delivery system with an acid‐sensitive group that could respond to internal stimuli efficiently.^[^ ^71^ ^]^ For instance, a pH‐responsive en‐srNP with pH‐cleavage amide bond and pH sponge PDEA was demonstrated to be size/charge changeable in an acidic environment.^[^ ^16^ ^]^ Through exploration of metabolism, distribution, and decomposition of the en‐srNPs at cellular and animal levels, such nano‐system showed great potential for synergistic photodynamic and PD‐L1 immunotherapy due to improved cancer penetration. In addition, imidazole, *N,N*‐dimethylaminoethyl methacrylate, maleic anhydride, methacrylic acid, and acrylic acid also belong to the pH‐sensitive monomers.^[^ ^72^ ^]^ The zeolitic imidazolate framework‐8 based on the pH‐responsive imidazole has been designed for controlled drug delivery.^[^ ^73^ ^]^ Meanwhile, many functional groups of polymers, such as benzoic imine bond, carboxylic acid, and phenylboronic acid, also show pH‐responsive transformations under different pH values.^[^ ^74^ ^]^ The pH‐mediated swelling behaviors caused by protonation have been regarded as principal factors in inducing the controlled deliveries of drugs like DOX or immune drugs.^[^ ^75^ ^]^ For instance, SCNs (SCNs/Pt) were encapsulated into the poly(ethylene glycol)‐*block*‐poly(2‐azepane ethyl methacrylate) embellished polyamidoamine (PAMAM) to form the Pt‐prodrugs.^[^ ^76^ ^]^ After accumulating in a weak acidity of cancer, PAEMA protonated and became hydrophilic SCNs/Pt into small nanoparticles for efficient cancer penetration.

Tumors always result in the dense extracellular matrix, aberrant vasculature, and elevate interstitial fluid pressure, all of which obstruct the effective accumulation and penetration of en‐srNPs into cancer cells.^[^ ^77^ ^]^ The studies demonstrated that smaller en‐srNPs showed better penetrability than larger ones, but particles with extremely low sizes suffered from insufficient cancer aggregation due to rapid clearance.^[^ ^78^ ^]^ To solve this dilemma, en‐srNPs maintained with suitable particle sizes are proposed to avoid fast clearance and transform into small particles for tumor penetration as they are docking at the tumor site.^[^ ^79^ ^]^ For example, polycaprolactone (PCL) homopolymer, PEG‐*b*‐PCL, and Pt prodrug linked poly(amidoamine)‐*g*‐PCL were self‐assembled into size‐convertible en‐srNP, also named as iCluster.^[^ ^80^ ^]^ In PAMAM/Pt prodrug, the drug was concatenated to the PCL end by 2‐propionic‐3‐methylmaleic anhydride as a pH‐labile linker. After the uptake by the cancer site, the cancer acidity cut off the pH‐linker and activated the separation of 5 nm diameter PAMAM/Pt. This rapid size conversion merit caused by pH‐responsiveness promoted accumulation and extravasation of nanoparticles for fast and effective cancer drug retention.^[^ ^81^ ^]^

**Figure 1:**
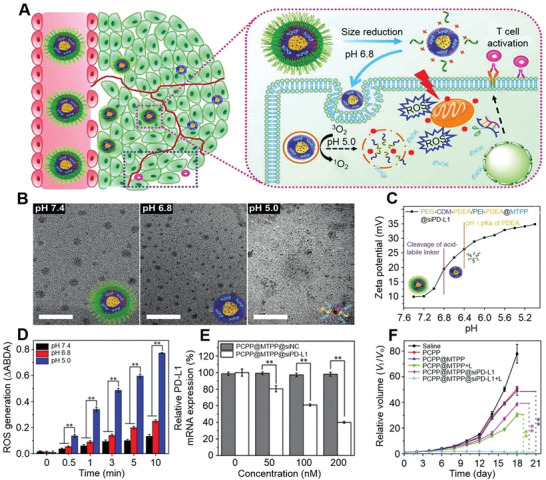
pH‐mediated en‐srNP for controlled drug release and synergistic cancer immunotherapy. A) Illustration of pH‐responsive dissociable PEG block connected poly(2‐(diethylamino) ethyl methacrylate)@PD‐L1‐targeting siRNA (PCPP@MTPP@siPD‐L1) micelleplex‐mediated photodynamic tumor immunotherapy in vivo. B) TEM images of PCPP@MTPP@siPD‐L1 after various treatments with pH 7.4, 6.8, and 5.0 for 4 h. C) Zeta potential variation of PCPP@MTPP@siPD‐L1 under various pH values. D) PCPP@MTPP@siPD‐L1 micelle‐induced ROS production in irradiation time‐ and acidity‐dependent manners. E) PCPP@MTPP@siPD‐L1 micelle releasing siPD‐L1 efficiency against PD‐L1 in B16F10 cells detected by qRT‐PCR. F) Relative tumor volumes after synergistic immunotherapy. Data are represented as mean ± standard derivation (SD; *n* = 6; *P < 0.05, **P < 0.01). Reproduced with permission.^[^ ^16^ ^]^ Copyright 2018, Wiley‐VCH.

Another dilemma for effective delivery of nanomedicine is its surface charge: Neutral or negative en‐srNPs, which tend to enhance blood circulation and cancer accumulation, thus cannot be effectively absorbed upon tumor cell endocytosis.^[^ ^82^ ^]^ Although positively charged en‐srNPs are beneficial for cellular endocytosis, they are easily cleared by the mononuclear phagocyte system during blood circulation.^[^ ^83^ ^]^ To solve these size effects and “charge dilemma”, size‐reduction and charge‐switch PCPP@MTPP@siPD‐L1 micelle with cancer acidity cleavable amide was designed (**Figure** **1**A). In the physiological microenvironment of pH 7.4, the pH‐responsive PCPP@MTPP@siPD‐L1 micelle remained neutral or negative charge to avoid clearance or interactions with nonspecific serum. Concurrently, as it was accumulating at the tumor tissue (pH 6.8), the PCPP@MTPP@siPD‐L1 micelle would be transferred to a small size and positive charge to improve tumor endocytosis further. After endocytosis by lysosomes (pH 4.5–6.0), the PEG block‐conjugated poly(2‐(diethylamino) ethyl methacrylate) core could be protonated quickly, resulting in the disassembly of carriers as well as enhanced drug release.^[^ ^16^ ^]^ As shown in Figure 1B,C, transmission electron microscopy (TEM) images proved the disassembly of PCPP@MTPP@siPD‐L1 micelle from 88 to 43 nm, and the zeta potential also indicated the charge transformation at weak acid as simulated TME.

For smart nanocarriers, a class of en‐srNPs with ultrasensitive surface charge and size‐switching capacity were utilized to load drugs for improving tumor therapy, which could be collapsed at low pH microenvironment of tumor in vitro or in vivo.^[^ ^84^ ^]^ The PCPP@MTPP system therapy effect was realized by combining near infrared ray (NIR) irradiation and ROS generation from 9,10‐anthracenediylbis(methylene) dimalonic acid (ABDA).^[^ ^16^ ^]^ As shown in Figure 1D, PCPP@MTPP en‐srNP generated significant ROS and showed a positive correlation with irradiation time and pH, which exhibited 4‐/5‐fold ROS production in pH 5.0 than pH 7.4. Briefly, acidic stimulus induced the dilapidation of vesicles and released the MTPP, which then recovered the photodynamic capability of MTPP. The effect of siPD‐L1 on PDT synergistic immunotherapy of PCPP@MTPP@siPD‐L1 in vitro was performed (Figure 1E). This phenomenon showed that the en‐srNP enhanced cancer penetration and endocytosis and reduced immune resistance by introducing immune checkpoint inhibitor drugs and initiating immune responses through tumor‐infiltrating T lymphocytes.^[^ ^85^ ^]^

These “all‐in‐one” en‐srNPs allow the design of multifunctional materials to strengthen the efficacy of synergistic cancer immunotherapy.^[^ ^86^ ^]^ Notably, tumor‐infiltrating cytotoxic T lymphocytes suppress tumor growth, but they are usually ineffective in the acid TME.^[^ ^87^ ^]^ The employments of systemically administrated en‐srNPs can modulate tumor acidity and reverse T cell activity, which are further combined with checkpoint blockade agents to realize effective immunotherapy.^[^ ^88^ ^]^ Therefore, the neutralizing tumor acidity increases recruiting of immune T cells or natural killer cells and reduces the immunosuppressive immune cells. Moreover, the en‐srNP could efficiently deliver RNA genes to cancer cells and block the checkpoint gene expression.^[^ ^89^ ^]^ As shown in Figure 1F, NIR irradiation of PCPP@MTPP@siPD‐L1 group activated a significant cancer growth inhibition owing to the release of siPD‐L1 for combination therapy (Figure 1F).^[^ ^16^ ^]^ Du et al. also proposed that the cathepsin/pH hierarchical‐responsive en‐srNP improved caner accumulation and synergistic immunotherapy, which thoroughly ablated cancers and inhibited cancer recurrence simultaneously.^[^ ^11b^ ^]^ Many pieces of research also demonstrated that sensitive polymers containing tertiary amine exhibited super pH‐responsive structural transformations under the stimulation of cancer acidity, which reached completion within seconds.^[^ ^90^ ^]^ This super‐pH‐sensitive en‐srNP further impaired TME and promoted tumor synergistic immunotherapy by improving the accuracy of drug release.^[^ ^91^ ^]^

### 2.2 GSH‐Mediated en‐srNPs

**Figure 2:**
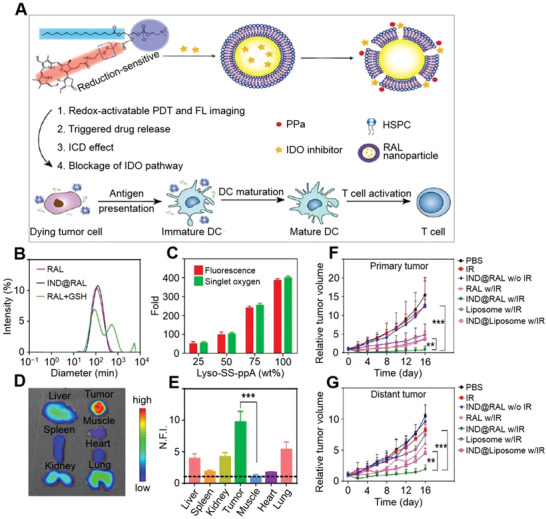
GSH‐mediated en‐srNP for controlled drug release and synergistic cancer immunotherapy. A) Schematic illustration of combined PDT and immunotherapy by IDO inhibitor loaded IND@RAL for combating cancer. B) DLS analysis of RAL, IND@RAL, and IND@RAL exposed to 10.0 mM GSH. Scale bar: 100 nm. C) Redox‐activatable fluorescence and ^1^O_2_ turn‐on behaviors of various liposomes with different percentages of redox‐sensitive lipids. D) Fluorescence imaging of collected organs at 24 h post‐injection of RAL‐PPa. E) Normalized fluorescence signal of organs to that in muscle in (D). ***P < 0.001, compared with muscle. F,G) Growth curves for primary and distant tumors of 4T1 tumor‐bearing mice after PDT treatment. Mice are irradiated with 660 nm laser with a power density of 400 mW cm^−2^ for 10 min after 24 h post‐injection at an equal PPa and IND dose of 3.0 and 2.5 mg (kg BW)^−1^, respectively. **P < 0.01, compared with IND@liposome with irradiation group. ***P < 0.001, compared with IND@RAL without irradiation group. Reproduced with permission.^[^ ^25^ ^]^ Copyright 2019, American Chemical Society.

TME is characterized as redox‐heterogeneous due to different GSH distributions in the intracellular environment of about 2.0–10.0 mM and extracellular matrix of about 2.0–20.0 µM.^[^ ^92^ ^]^ GSH‐responsive nanocarriers are designed to achieve good stability in an extracellular microenvironment with low GSH levels. Rapid response to the intracellular high GSH level can directly deliver drugs into the nucleus and cytosol.^[^ ^93^ ^]^ The designed micelles of GSH‐mediated en‐srNPs are generally achieved by the S─S or Se─Se bond in the hydrophobic backbone.^[^ ^94^ ^]^ As shown in **Figure** **2**A, amphiphilic polymer blocks with S─S bond were self‐assembled into en‐srNP with an encapsulation of indoleamine 2,3‐dioxygenase (IND) inhibitor.^[^ ^25^ ^]^ After incubating with 10.0 mM of GSH, a rapid release of drugs was triggered upon the disruption of liposomes. Another approach to produce GSH‐mediated en‐srNPs was conjugating CPT or PTX chemotherapeutics as hydrophobic ends with hydrophilic ends via S─S or Se─Se links.^[^ ^95^ ^]^ As expected, the disulfide bonds of en‐srNPs were readily broken by the high GSH level in tumor cells to achieve targeted drug delivery and reinforce intracellular redox reactions.

A self‐reinforcing chemodynamic therapy (CDT) nanoagent based on MnO_2_ was reported to have properties of releasing Fenton‐like metal ions and GSH depletion.^[^ ^96^ ^]^ This strategy is dependent on GSH generation in situ, which is a distinct advantage of designed en‐srNPs for synergistic immunotherapy.^[^ ^97^ ^]^ The mesoporous silica‐coated by MnO_2_ (MS@MnO_2_ NP) underwent a redox with GSH, obstructing the tumor defense system and self‐reinforcing CDT.^[^ ^98^ ^]^ Upon uptake of MS@MnO_2_ NP by cancer cells, burst drug release was achieved through the degradation of MnO_2_ shell. Simultaneously, the endogenous GSH and hydrogen peroxide (H_2_O_2_) were decomposed for impairment of the antioxidant defense system (ADS) and relieved tumor hypoxia. Together with anti‐tumor drugs, en‐srNP with Fenton reactions achieved CDT self‐reinforcing combination therapy.^[^ ^99^ ^]^ Other approaches incorporating GSH‐responsive crosslinking agents based on Cu^2+^, Fe^3+^, and Pt were proposed to destruct TME and activate synergistic therapy.^[^ ^100^ ^]^ Ling et al. developed an en‐srNP by self‐assembling lipid‐PEG with resisting thiol‐mediated detoxification to deliver Pt (IV) prodrugs.^[^ ^101^ ^]^ The oxidation of such prodrugs disrupted the hydrophilic/hydrophobic balance, leading to the collapse of water‐soluble monomers and subsequent drug release. Interestingly, the decomposed metal ions acted as contrast agents for imaging monitoring, which represented an attractive route for developing multi‐modal anti‐tumor theranostics.^[^ ^102^ ^]^

For nano‐systems, their shape and structure of en‐srNPs are essential for tumor endocytosis. The dynamic laser scattering (DLS) analysis showed that the IND‐loaded redox‐activatable liposomes (IND@RAL) were 90 nm on average diameter.^[^ ^25^ ^]^ Moreover, the structure of the IND@RAL was disassembled after adding 10.0 mM GSH, indicating an on‐demand release of drugs in a unique microenvironment by the breakage of the disulfide bond (Figure 2B). In addition, different amounts of GSH‐sensitive linker and porphyrin‐lipid were conjugated in the IND@RAL, and the production of singlet oxygen (^1^O_2_) increased up to 400‐fold by the increment of porphyrin‐lipid and redox‐responsive bond (Figure 2C). Xu et al. also reported that en‐srNP based on cisplatin could respond to GSH to achieve an efficient drug release, which synergistically amplified ROS and eventually increased the concentration of H_2_O_2_ and high toxicity ROS like hydroxyl radicals (•OH).^[^ ^103^ ^]^ After GSH‐mediated en‐srNPs accumulation, the tumor was converted to fragility, and the drugs were released to promote synergistic immunotherapy.^[^ ^104^ ^]^

The high intracellular GSH consuming level could strengthen PDT activity after endocytosis of en‐srNPs by cancer cells, thus efficiently eliminating cancer growth.^[^ ^105^ ^]^ For example, the Janus nano‐bullets integrating Ce6 and magnetic heads with disulfide‐bridged mesoporous organosilica bodies were proposed for redox/pH‐triggered PS release.^[^ ^22^ ^]^ The up‐regulation of ROS elicited immunogenic cell death (ICD) response was dependent on mechanisms of autophagy, endoplasmic reticulum stress, and apoptotic cell death. In this Janus nano‐bullet, magnetic hyperthermia (MHT) and PDT simultaneously elicited a sequence of ICD, resulting in synergistic tumor‐specific immune responses. This designed GSH‐mediated en‐srNP in combination with various drugs exhibited efficient therapy for cancer.^[^ ^106^ ^]^

These GSH‐mediated en‐srNPs showed precise drug selective release and synergistic immunotherapy. As shown in Figure 2D, the fluorescence images revealed that the GSH‐targeted IND@RAL accumulated and displayed stronger fluorescence after 3 h at the cancer site compared to the normal organs, which proved to have an excellent specific accumulation.^[^ ^25^ ^]^ In addition, the quantitative fluorescence signal of IND@RAL in the tumor site was nearly 10‐fold higher than the adjacent muscle (Figure 2E). After uptake by cancer cells, such GSH‐mediated IND@RAL burst released the multi‐drugs to strengthen synergistic therapy. In addition, the MnO_2_‐ and PTX‐loaded en‐srNP also showed excellent stability and ability to localize tumor sites that were monitored by “on−off” fluorescent switch.^[^ ^107^ ^]^ Therefore, the en‐srNP has been designed to deliver drugs effectively to cancers and achieve high loading of the multifunctional drugs.^[^ ^108^ ^]^

The effect of GSH‐mediated IND@RAL synergizing immunotherapies against primary or distant tumors was assessed by prior works.^[^ ^25^ ^]^ As shown in Figure 2F,G, IND@RAL did not inhibit either primary or distant tumors without photo irradiation. The RAL group with irradiation inhibited primary cancers. However, it could not significantly control remote cancer. On the contrary, the IND@RAL with photo‐irradiation induced obvious growth inhibition on distant and primary cancers compared to the non‐redox activatable liposomes, indicating that synergistically PDT‐mediated photo‐toxicity and IDO inhibitory treatment reduced the rate of tumor growth. The biomimetic en‐srNP activated the immune system and enabled the significant elimination of distant and deep cancers, which helped develop advanced synergistic immunotherapy to defeat metastasis tumors.^[^ ^109^ ^]^

GSH‐mediated en‐srNPs have been widely explored and developed in tumor synergistic immunotherapy to kill metastatic tumors and set a long‐term tumor memory.^[^ ^110^ ^]^ These en‐srNPs are designed to activate drug release, reverse TME, encapsulate immune drugs, and enhance cascade therapy activity (>100‐fold).^[^ ^25^ ^]^ For instance, integrating oxaliplatin (OXA), photosensitizer, and CD47 blockade antibody into one en‐srNP efficiently inhibited the primary and remote cancer growth in the bilateral model.^[^ ^111^ ^]^ Combining Fenton catalysts, GSH depletion agent, and DOX with checkpoint inhibitors launched a vigorous activity of anti‐remote cancers. While integrating cyclic dinucleotide and STING‐activating en‐srNP improved the efficiency of home cancer therapy and the clinical outcome of immunotherapy.^[^ ^112^ ^]^

### 2.3 Enzyme‐Mediated en‐srNPs

Enzymes play an important role in nanomedicine due to their excellent catalytic properties and bio‐recognition capabilities. The enzyme reactions observed in all pathological, physiological, and metabolic processes are efficient and selective, which have been employed as responsive modules for designing en‐srNPs.^[^ ^113^ ^]^ Therefore, enzyme‐mediated en‐srNPs for controlled drug delivery are cataloged by the effector biomolecule, such as proteases, lipases, hydrolases, oxidoreductases, and glycosidase.^[^ ^114^ ^]^ For example, the core‐shell ICG/DOX@Gel‐CuS en‐srNP was consisted of CuS en‐srNP and gelatin (Gel) en‐srNP with loading DOX/ICG.^[^ ^115^ ^]^ After accumulating ICG/DOX@Gel‐CuS en‐srNP, cancer overexpressing enzymes activated the disassembly of Gel and controlled delivery of ICG/DOX, which were visually imaged by the increasing fluorescent signal in the cancer field.

The response mechanism of en‐srNPs was considered as the imbalance of hydrophilic and hydrophobic materials comprised of enzyme‐responsive moieties.^[^ ^116^ ^]^ The quinone oxidoreductase 1 (NQO1) enzyme‐mediated en‐srNP was designed by self‐assembling amphiphilic BCPs, coumarin photosensitizer, and quinone trimethyl enzyme‐sensitive block linkages.^[^ ^117^ ^]^ Upon the NQO1 micelle endocytosis, the cancer NQO1 enzyme triggered the breakage of quinone and controlled delivery of linked photosensitizer and then generated PDT activity and fluorescent emission. In addition, the immune drugs encapsulated in the enzyme‐mediated en‐srNPs avoided systemic circulation and thus decreased off‐target side effects.^[^ ^118^ ^]^ Immunotherapy combined with enzyme‐mediated en‐srNPs targeted and localized within the enzyme environment, where they supported immune cells and counteracted the immunosuppressive microenvironment by awaking T cells activity.^[^ ^119^ ^]^

**Figure 3:**
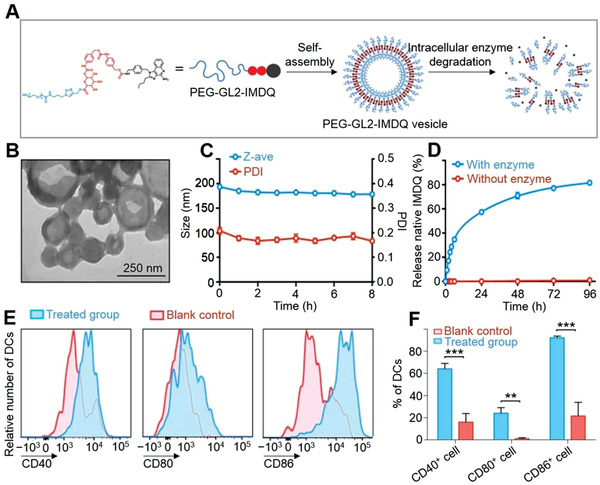
Enzyme‐mediated en‐srNP for controlled drug release and synergistic cancer immunotherapy. A) Self‐assembly of PEG‐modified glucuronide (GL2) linker IMDQ amphiphile prodrug into PEG‐GL2‐IMDQ micelle. B) TEM image of self‐assembled en‐srNP. C) Stability of PEG5k‐GL2‐IMDQ en‐srNP after dilution in phosphate‐buffered saline (PBS) at pH 7.4 and incubated at 37 °C (*n* = 3). D) In vitro release of IMDQ from PEG5k‐GL2‐IMDQ vesicle in the presence of an esterase at pH 5.0 and 37 °C (*n* = 3). E) Flow cytometry analysis for expression of maturation markers on DCs in the draining lymph node in response to subcutaneous injection of PEG5k‐GL2‐IMDQ en‐srNP. F) Percentages of DCs expressing a specific maturation marker (Student *t*‐test; *n* = 3; **P < 0.01, ***P < 0.001). Reproduced with permission.^[^ ^28^ ^]^ Copyright 2019, American Chemical Society.

Enzyme‐mediated prodrug en‐srNPs exhibit excellent blood circulation, high drug loading rate, and tumor targeting accumulation.^[^ ^120^ ^]^ As shown in **Figure** **3**A, the prodrug of PEG‐GL2‐IMDQ micelles, including two benzyl blocks and PEG amphiphile, was self‐assembled with an enzyme‐responsive effect.^[^ ^28^ ^]^ After uptake by APCs, PEG‐GL2‐IMDQ micelle was degraded, and imidazoquinoline (IMDQ) was released, thus combining to toll‐like receptors (TLR) 7 and 8 for inducing robust immune activity. Compared to non‐controlled drug delivery of TLR agonists, locally administered PEG‐GL2‐IMDQ micelles in vivo provoked lasting immune respond in draining lymphoid tissue and interferon expression. In addition, enzyme‐responsive tide linkers were also fabricated to control drug delivery.^[^ ^121^ ^]^ Once the en‐srNP was accumulated in the tumor region, effector proteases were consumed and finally resulted in a burst local drug release of prodrug en‐srNP.

As shown in Figure 3B,C, PEG‐GL2‐IMDQ micelle self‐assembled into en‐srNP was observed with a hollow structure (195 nm, PDI = 0.2) by DLS and TEM.^[^ ^28^ ^]^ The structure of PEG‐GL2‐IMDQ micelle was disassembled after the addition of enzyme due to the cleavage of the GL2 linker for controlled drug delivery. As shown in Figure 3D, the low IMDQ release was observed in the absence of enzymes, but the quantitative release of IMDQ attained a peak for five days in the cancer enzyme microenvironment. The PEG‐GL2‐IMDQ micelle activated DCs, which were the specialized APCs and a target T cell in vaccination and immune therapy. After injection of PEG‐GL2‐IMDQ micelle, flow cytometry analysis of the draining lymph node showed a strong increasing number of DCs (fivefold), manifesting the ability of the en‐srNP to recruit DCs (Figure 3E,F). Analysis of maturating DC markers (CD86, CD40, and CD80) manifested a significant maturation of DC T cells by PEG‐GL2‐IMDQ micelle compared to those without treatment. In addition, the PEGylated dendritic polymer‐DOX prodrug with a multi‐stimuli sensitivity was designed as a controlled drug delivery platform.^[^ ^31^ ^]^ The breakage of hydrolyzing hydrazone bond between copolymer and DOX framework induced DOX release in acid cancer cells.

Many well‐designed new tumor immunotherapy strategies, for example, angelica sinensis increased the expression of IL‐2 and decreased the expression of IL‐10, which have improved survival rates in cancer therapy.^[^ ^122^ ^]^ The combination of immunotherapy and improvement of TME provides an effective and safe synergistic strategy for the intervention of tumors. The microenvironments of enzymes play crucial roles in evading immune surveillance and offer favorable conditions for the progression of malignant cancers.^[^ ^123^ ^]^ Smart enzyme‐mediated en‐srNPs predictively and selectively reacted with enzymes, leading to target delivery of immune drugs for improved therapeutic effect.^[^ ^124^ ^]^ Li et al. summarized enzyme‐mediated en‐srNP including cleavable/uncleavable linker, hydrophilic crown, and targeting ligand for immune drug delivery.^[^ ^125^ ^]^ As such, immunotherapy that targets and localizes within the tumor cells shows excellent potential as a promising anti‐tumor treatment because it can support immune cells and counteract the immunosuppressive microenvironment. The enzyme‐mediated delivery of immune drugs maintains drug efficacy, decreases immune toxicity, and provides a platform for synergistic cancer immunotherapy.

### 2.4 ROS‐Mediated en‐srNPs

Due to the overexpression of superoxide dismutase in the tumor cells, cancers tend to produce a high level of intracellular ROS, which offers an opportunity to fabricate intelligent en‐srNPs for specific tumor therapy with ROS‐responsive.^[^ ^126^ ^]^ Among all ROS species in tumor cells, H_2_O_2_ is one of the most stable and abundant ROS. As a result of the large surface area, high reactivity, and small size, en‐srNPs act as an H_2_O_2_ catalyst for generating O_2_ and releasing tumor hypoxia environment.^[^ ^127^ ^]^ In addition, the metal compound nanoparticles, such as copper, gold, and manganese, can decompose H_2_O_2_ to •OH by Fenton‐like or Fenton reactions.^[^ ^128^ ^]^ The en‐srNPs stay as stable constructions in the normal tissue microenvironments and undergo chemical and physical changes as they are accumulated in cancer cells.^[^ ^129^ ^]^ Wu et al. demonstrated that biocompatible Fe(III) species‐WS_2_‐polyvinylpyrrolidone nanocapsule owned an excellent DOX loading and •OH capacity.^[^ ^130^ ^]^ Because of the strong reactivity of •OH, such en‐srNP had excellent potential for killing cancer cells via various mechanisms, such as upgrading PTT, enhancing PDT, improving CT, and/or directly killing tumor cells.^[^ ^131^ ^]^

Many reactive chemical species, for example, H_2_O_2_, superoxide radical, and •OH, are elevated in the cancer cells. Hence, the consumption of intracellular redox microenvironment via upregulating and/or downregulating ROS strengthens intracellular oxidative stress for effective cancer treatment.^[^ ^132^, ^133^ ^]^ Fenton reactions utilize Fe^2+^ in situ as a catalyzer to transfer H_2_O_2_ to •OH, which was studied for its capacity of killing tumor cells by CDT. The ferrocene and its derivatives with Fe^2+^ molecule show prospect as the nano‐catalytic chemo‐dynamic agents to enable the disproportionation of H_2_O_2_ and the creation of •OH.^[^ ^134^ ^]^ By using endogenous H_2_O_2_, most cancers can be eliminated to boost CDT by the Fenton‐like reaction.^[^ ^135^ ^]^ PDT has an advantage among various therapy strategies since it uses minimally invasive treatment and non‐invasive character for various malignant tumors. Tumor hypoxia is the “Achilles' heel” of the traditional PDT.^[^ ^136^ ^]^ PDT consuming O_2_ would aggravate cancer hypoxia, thus potentially resulting in many negative results, for example, tumor metastasis, tumor invasiveness, and malformation angiogenesis.

**Figure 4:**
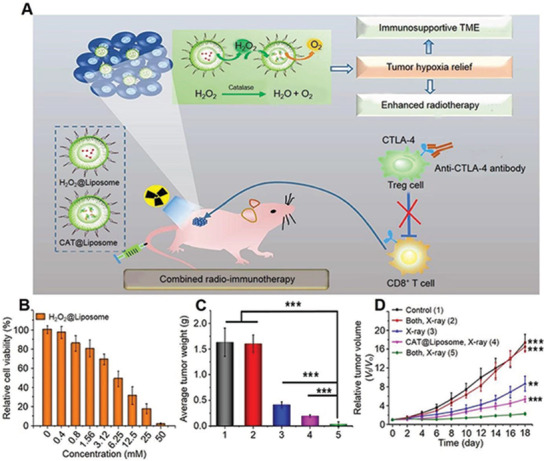
ROS‐mediated en‐srNP for controlled drug release and synergistic cancer immunotherapy. A) Schematic illustration of H_2_O_2_@Liposome and catalase (CAT@Liposome) for enhanced radio‐immunotherapy of cancer. B) Relative viability of 4T1 cells after incubation with H_2_O_2_@Liposome at various concentrations for 24 h. C) Average tumor weights measured on day 16 after different treatments. D) Tumor growth curves of mice after various treatments including, 1) Control, 2) Both, X‐ray−. 3) X‐ray+, 4. CAT@Liposome, X‐ray+, 5. Both, X‐ray+. Data are represented as mean ± SD (two‐tailed *t*‐test; *n* = 5; *P < 0.05, **P < 0.01, and ***P < 0.001). Reproduced with permission.^[^ ^133^ ^]^ Copyright 2018, American Chemical Society.

To overcome these deficiencies, many studies are centralized on catalyzing H_2_O_2_ to O_2_ for relieving the tumor hypoxia and thus enhancing PDT efficacy. Combining the O_2_ generation and strengthened PDT activity through NIR and a water‐soluble photosensitizer was imperative to achieve the self‐promoted enhancement of PDT.^[^ ^137^ ^]^ Up to now, several H_2_O_2_‐activated or H_2_O_2_‐dependent nano‐systems have been reported for ameliorating cancer hypoxia, enhancing PDT, and imaging. For example, a unique multilayer structure coated with MnO_2_ reacted with endogenous acidic H_2_O_2_ to elevate the dissolved O_2_ concentration, enhancing cancer therapy efficacy.^[^ ^138^ ^]^ The productions of Mn^2+^ and O_2_ were considered as Fenton reagents and hypoxia release for magnetic resonance imaging (MRI) and synergistic therapy, respectively. The relief of cancer hypoxia relieved the limitation of PDT and the low immune responses.^[^ ^129^ ^]^ Meanwhile, the overcoming of tumor hypoxia increased drug sensitivity by reducing p‐glycoprotein expression (**Figure** **4**A). In addition, many research have also explored that releasing of cancer hypoxia could decrease the immune‐suppression to the cancers via the activation of tumor‐associated macrophages.^[^ ^139^ ^]^

The tumor cell proliferation process requires extra energy and nutrient supplement. The upregulation of aerobic glycolysis named the “Warburg effect” influences tumor cell growth by changing glucose concentrations.^[^ ^140^ ^]^ A new strategy was designed to improve therapy efficiency by cutting off the tumor glucose supplement and destroying the glucose metabolism elements, which selectively inhibited tumor cells.^[^ ^141^ ^]^ On the other hand, upregulated endogenous H_2_O_2_ within cancer cells is still insufficient for effective reactions. Therefore, various H_2_O_2_ generating agents, for example, glucose oxidase, cisplatin, and vitamin C, have been explored to deplete glucose and elevate H_2_O_2_ concentration in tumor cells.^[^ ^142^ ^]^ Feng et al. reported a type of magnetic nanoparticle, where the inner glucose oxidase was encapsulated with a high loading enzyme, and MnO_2_ nano‐shell was designed as an intelligent “gatekeeper” shield.^[^ ^143^ ^]^ It was found that the cascade reactions promoted the catalysis of glucose to H_2_O_2_, decomposing H_2_O_2_ to O_2_ and enhancing the production of ^1^O_2_ upon NIR irradiation. Hence, the synergistic PDT and starvation therapy magnified by cascade reactions efficiently inhibited the tumor in a spatiotemporally controlled manner.

Even at low concentrations, single H_2_O_2_ exhibited a significant effect on killing cancer cells, but H_2_O_2_@Liposome had a relatively low toxicity.^[^ ^133^ ^]^ Meanwhile, the cytotoxicity was dramatically enhanced at high H_2_O_2_@Liposome concentrations (Figure 4B). As shown in Figure 4C,D, the most potent cancer suppression effect was observed in the group of H_2_O_2_@Liposome plus CAT@Liposome therapy, in which the cancer therapy efficacy was the highest compared to RT alone or RT enhanced by CAT@Liposome owing to the cascade reactions. Yao et al. developed an up‐conversion hollow mesoporous cerium oxide bio‐photo‐catalyst for H_2_O_2_‐responsive O_2_‐consuming tumor treatment.^[^ ^144^ ^]^ Synergistic anti‐tumor efficiency of the assembled en‐srNP was enhanced in the bearing cancer mice.^[^ ^145^ ^]^ Ke et al. reported en‐srNP with Fe^2+^ and H_2_O_2_ Fenton reaction to produce •OH, and the controlled drug delivery was specifically activated for efficiently ablating cancer cells.^[^ ^146^ ^]^ In summary, systemic injection of en‐srNP with specially released hypoxia is an effective and safe approach to enhance cancer treatment dramatically.^[^ ^147^ ^]^

Recently, a number of signs of progress have been achieved for transforming TME into an unfavorable environment for cancer growth.^[^ ^148^ ^]^ Many pieces of research indicate that H_2_O_2_ can upregulate cell division and proliferation gene transcription, facilitate cancer cells to infiltrate normal tissues, and enhance the viability of cancer cells.^[^ ^149^ ^]^ Therefore, cancer cells are more sensitive than normal tissues and do not bear either excessively increased or decreased H_2_O_2_ levels to induce dead tumors. Chang et al. fabricated the hollow‐structured Cu_2_MoS_4_ en‐srNP bioreactor for synergetic CDT, PDT, immunotherapy, and starvation therapy of tumors.^[^ ^150^ ^]^ These ROS‐mediated en‐srNP strategies can be further developed for specific tumor therapies, such as improving CT, upgrading PTT, enhancing PDT, synergistic immunotherapy, and directly destroying cancer cells.^[^ ^151^ ^]^

## 3 Exo‐Stimuli‐Responsive Nanoparticles (ex‐srNPs) for Smart Drug Release and Synergistic Cancer Immunotherapy

### 3.1 Photo‐Mediated ex‐srNPs

Advances in nanotechnology and nanoscience lead to the occurrence of several new cancer therapy strategies due to their unique physicochemical properties, including photo‐induced drug delivery systems, PTT, PDT, and synergistic immunotherapy.^[^ ^152^ ^]^ Under the local NIR laser irradiation, the photo‐cleavage coumarin‐containing and o‐nitrobenzyl are broken for controlled drug release and synergistic therapy.^[^ ^153^ ^]^ Chen et al. designed a kind of micelles that was composed of cleavable hydrophobic poly(4,5‐dimethoxy‐2‐nitrobenzyl methacrylate) (PNBMA) compounds.^[^ ^154^ ^]^ Based on NIR activation of the up‐conversion nanoparticles (UCNPs), the hydrophobic photosensitive PNBMA parts had a hydrophobic to hydrophilic transition, thereby triggering the controlled drug delivery of AB3 inhibitor. Such photothermal‐sensitive polymeric nanocarrier also exhibited particular advantages for releasing drugs via the change of temperature.^[^ ^155^ ^]^ Besides, hyperthermia disintegrated ex‐srNP structure and enhanced tumor‐specific cellular uptake, leading to a synergetic chemo/PTT effect for metastatic cancer therapy.^[^ ^156^ ^]^

PDT destructs tumor cells by generating ^1^O_2_, which is produced via a photodynamic reaction of the photosensitizer under a specific excitation wavelength.^[^ ^157^ ^]^ Owing to the production of ^1^O_2_ coming from O_2_, most PDT strategies belong to the oxygen‐dependent type. Several studies summarized two possible approaches to alleviate tumor hypoxia microenvironment: the delivery of O_2_ to the cancer site or generating O_2_ in situ under cancer hypoxia.^[^ ^158^ ^]^ Consequently, different ex‐srNPs are designed to contain O_2_ carriers (e.g., hemoglobin, perfluorocarbon) and oxygen generators (e.g., MnO_2_).^[^ ^159^ ^]^ Based on O_2_ supplement, PDT provides a high specificity for treating cancers by controlling the photo exposure at specific positions for decreasing cytotoxicity and side effects.

PDT, PTT, and many CT drugs directly eliminate cancers and produce ICD through releasing TAAs to activate an immune response.^[^ ^161^ ^]^ Compared to PDT and CT, PTT holds many superiorities, such as negligible system side effects, no reliance on the O_2_, and without drug resistance compared with CT.^[^ ^163^ ^]^ Furthermore, the PTT increasing permeability and blood circulation contributes to internalization and deep penetration.^[^ ^162^ ^]^ Based on the precisely controlled NIR laser in the local tumor, TAAs are released as a powerful support to inefficient antigens in situ. The photo‐responsive IR820−1MT NP was demonstrated with significantly enhanced accumulations of helper T cells and cytotoxic T cells while inhibited amounts of regulatory T cells.^[^ ^163^ ^]^ Furthermore, the activated immune T cells attacked both primary and metastatic tumors and prevented tumor recurrence by a systemic anti‐tumor immune response and memory T cells.^[^ ^164^ ^]^ Therefore, the combination of “hot” immunogenic TME, an immune checkpoint inhibitor, and various drugs for synergistic tumor therapy in the all‐rolled‐into‐one ex‐srNP system showed high efficiency to inhibit cancer metastasis and deep cancer.^[^ ^165^ ^]^ Such a “vaccine‐like” in situ strategy is promising for patient‐specific antigens, and inner bed or deep solid cancers are short of immune cells infiltration and induce immune escape.

**Figure 5:**
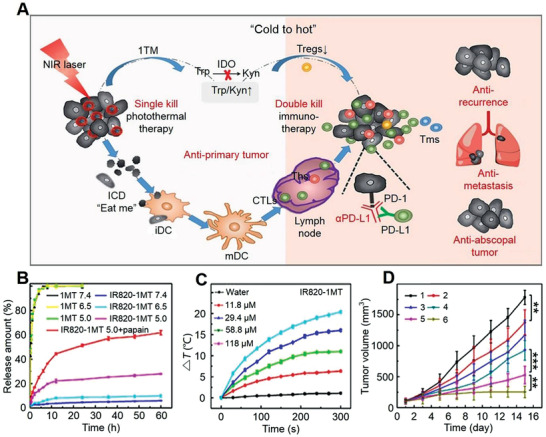
Photo‐mediated ex‐srNP for smart drug delivery and synergistic cancer immunotherapy. A) Schematic illustration for mechanism of IR820‐1MT NP (photothermal agent: IR820; immune checkpoint inhibitor: 1MT) to inhibit primary tumor and tumor metastasis and recurrence based on enhanced immunotherapy via synergistic PTT. B) In vitro 1MT release in various conditions. C) Temperature changes in organic photothermal agent (IR820‐1MT) nanoparticle solution after irradiation (1.0 W·cm^−2^, 660 nm). D) Change of average tumor volume after treatment with 1) normal saline, 2) 1MT, 3) αPD‐L1, 4) IR820, 5) IR820‐1MT, or 6) IR820‐1MT+αPD‐L1. Data are represented as mean ± SD (*n* = 9; *P < 0.05, **P < 0.01, ***P < 0.001). Reproduced with permission.^[^ ^160^ ^]^ Copyright 2019, American Chemical Society.

As shown in **Figure** **5**A, the IR820−1MT NP was a type of cascade activating nano‐platforms by converting “cold” to “hot” TME via the NIR‐triggered domino effects and immune drug release.^[^ ^160^ ^]^ The hydrophobic 1MT conjugated to the hydrophilic IR820 and formed the amphiphilic IR820−1MT molecule, which were self‐assembled to IR820−1MT NP without any sediment in the solution of deionized water. The obtained IR820−1MT NP was stable for at least three months. Furthermore, IR820−1MT NP released only 5.9% of 1MT for 60 h in PBS, showing high stability. On the contrary, 27.8% of 1MT drugs were controlled release by studying IR820‐1MT NP in pH 5.0 PBS for 60 h, indicating an excellent pH‐responsive controlled drug delivery behavior (Figure 5B). Chu et al. also designed NIR‐photo‐responsive UCNP immune devices for selectively triggering anti‐tumor immunotherapy, exhibiting extraordinary tumor inhibition and anti‐metastasis efficacy.^[^ ^166^ ^]^

To verify the photothermal character, IR820‐1MT ex‐srNP was irradiated with NIR based on the maximum absorption peak.^[^ ^160^ ^]^ As shown in Figure 5C, the temperatures of IR820−1MT NP solution increased by 20.4 °C and the single IR820 solution increased by 22.2 °C under irradiating at the same concentration, indicating potent hyperthermia. The photo‐mediated IR820−1MT NP could be applied alone or with synergistic immunotherapy. The immune drug of αPD‐L1 loaded by IR820−1MT NP contained two or more therapy strategies. As shown in Figure 5D, the IR820−1MT NP loaded with αPD‐L1 greatly enhanced the therapy efficacy, such as an inhibition ratio of 87%.^[^ ^160^ ^]^ The designed IR820−1MT ex‐srNP exhibited high efficacy toward inhibiting metastasis and recurrence of the tumor. In addition to the excellent capability for infrared thermal imaging, ex‐srNP possessed the high stability and capability of heat conversion.^[^ ^167^ ^]^ The enhanced hyperthermia was effective for protein unfolding and DNA denaturation to realize an efficient tumor therapy.

These controlled immunity regulations allowed the generation of effective immune responses in deep tumors, thereby maintaining long‐term anti‐tumor efficacy. Additionally, this ex‐srNP releasing cancer hypoxia that potentiated a cancer system immune response enhanced the synergistic therapeutic effect.^[^ ^168^ ^]^ The dying cancer cells and releasing “eat me” associated molecular signals for maturating dendritic cells and inducing robust effector cell generation synergistically killed the inner bed cancer and activated distant effects.^[^ ^169^ ^]^ Ma et al. designed phototherapy ex‐srNP that induced deep tissue ICD and potentiated cancer immunotherapy.^[^ ^170^ ^]^ Photo‐inducing synergistic immune therapies have emerged as another powerful therapeutic strategy via remote‐loading for simultaneous induction of ICD and reversing the immune‐suppressive TME.^[^ ^171^ ^]^ As expected, the CD^8+^/CD^4+^ and other immune cells were activated, and the secretion cytokines (IL‐12 and TNF‐α) were further enhanced. In this context, the *co*‐stimulation of regulating T cells could activate cancer‐specific immune responses, especially for treating mid and late‐stage tumor patients synergistically.^[^ ^172^ ^]^

### 3.2 Ultrasound‐Mediated ex‐srNPs

The US plays a vital role in many biomedical applications. For example, the US at high power can be employed for eliminating cancer cells, while the US at low power is generally useful for imaging and diagnosing the cancer position.^[^ ^173^ ^]^ Three main criteria are essential for the design of US‐mediated ex‐srNPs: 1) Reliable and stable drug encapsulation; 2) Responsiveness to special US power; 3) The ability of imaging‐controlled drug delivery for synergistic cancer therapy. Several mechanical and thermal effects, including acoustic fluid streaming, local hyperthermia, cavitation, and pressure variation, induce the penetration of US‐mediated ex‐srNPs upon US waves radiation through the body.^[^ ^174^ ^]^ Benefiting from the textural properties, such as high biocompatibility and cavity volume, US‐mediated ex‐srNPs offer a platform with high penetration ability for different loading drugs.

**Figure 6:**
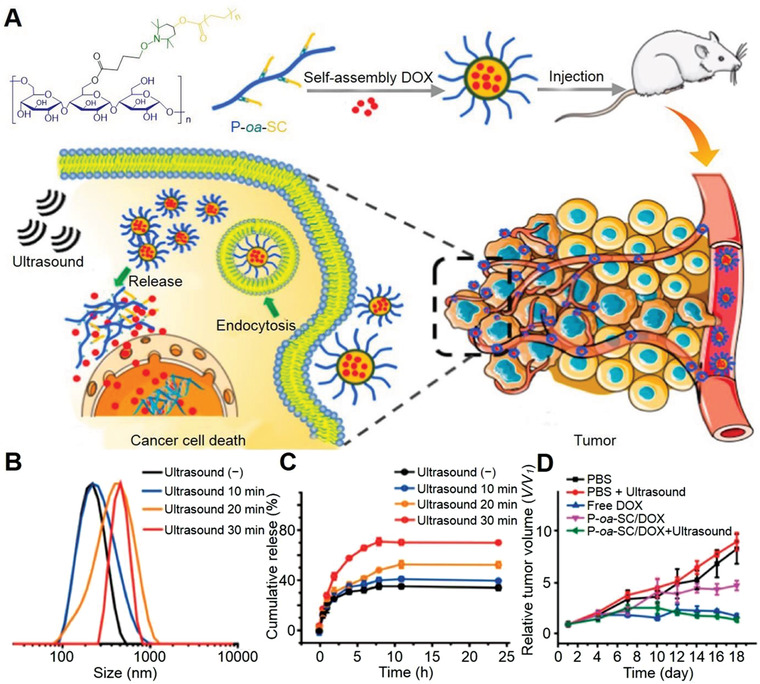
US‐mediated ex‐srNP for smart drug delivery and synergistic cancer immunotherapy. A) Illustration of construction of US‐responsive pullulan‐based amphiphilic polymer with multiple hydrophobic stearic segments through US‐labile linkage of P‐*oa*‐SC NP to pursue US‐specified chemotherapeutic potency to the tumors. B) DLS measurement for the P‐*oa*‐SC self‐assembly upon ultrasonication (1.0 MHz, 9.9 W, 3 W cm^−2^). C) The cumulative release of DOX from P‐*oa*‐SC/DOX in the presence of US impetus (1.0 MHz, 9.9 W, 3 W cm^−2^). Data are represented as mean ± SD (*n* = 3). D) Tumor growth profile. Data are represented as mean ± standard error (S.E.). *P < 0.05. Reproduced with permission.^[^ ^176^ ^]^ Copyright 2018, American Chemical Society.

Precision cancer therapies request a preferable transport of the medicine to the specific sites and release of relative treatment drugs to the cancer location in time.^[^ ^175^ ^]^ As shown in **Figure** **6**A, the US‐labile oxyl‐alkylhydroxylamine was employed as oxyl‐alkylhydroxylamine bond nanoparticle (P‐*oa*‐SC NP) linkages between hydrophobic stearic segment and the hydrophilic pullulan.^[^ ^176^ ^]^ The average diameter of P‐*oa*‐SC NP was observed to follow a consistent rise from 202.80 to 633.30 nm with the prolonged US for 30 min (Figure 6B). Upon US impetus, the prepared P‐*oa*‐SC NP exhibited distinct structure collapse and presented the controlled release of the drugs. In the presence of US, the drug release rate, approximately 32% release within 1 h, has markedly accelerated (Figure 6C). In addition, the P‐*oa*‐SC/DOX group exhibited distinct suppression of cancer growth with eliciting DOX release in tumor site to impart chemotherapeutic efficacy.

The US induces an oscillating pressure between the inner and surface cell membrane, thus producing membrane pores to strengthen drug penetration within the cancer cell membrane.^[^ ^177^ ^]^ In addition, the drug‐loading ex‐srNPs are generally decorated by employing internalization ligands (targeting agents) for active target or passive target. Zhou et al. designed US‐responsive ex‐srNP with targeting group to tumor, incorporating a lipophilic sono‐sensitizer of Ce6 to the liposome.^[^ ^47^ ^]^ Upon irradiation in local cancer tissue by the US, the Ce6 creating SDT induced an effective liposome disruption. Hence, the breakdown of lipid bilayer by US triggered a controlled delivery of DOX, which then combined US‐mediated CT and SDT synergistically. Overall, the accumulation of US‐mediated ex‐srNPs inhibited cancer growth.

SDT can also consume the O_2_, and the hypoxic TME is further exacerbated, which is not conducive to treating tumors.^[^ ^178^ ^]^ Zhu et al. reported that the MnO*_x_* component could be utilized as an inorganic nano‐enzyme to convert the cancer H_2_O_2_‐overexpressed molecules to O_2_ and thus modify hypoxia TME.^[^ ^179^ ^]^ Upon US irradiation, the activated sono‐sensitizers generated the toxic ^1^O_2_ by transferring energy to neighbor O_2_ for cancer therapy. Like photosensitizers for PDT, many organic compounds have been employed as SDT agents, including but not limited to phthalocyanines, porphyrin, and porphyrin derivatives.^[^ ^180^ ^]^ Nonetheless, these organic sono‐sensitizers are limited by low bioavailability and easy clearance out of the body. To solve these deficiencies, many nanocarriers (e.g., mesoporous silica, graphene, phospholipid microbubble, and polymer microbubble) are utilized to enhance the synergistic SDT effect.^[^ ^181^ ^]^

Paris et al. designed a hierarchical US‐mediated smart mesoporous silica nanocarrier for tumor treatment.^[^ ^182^ ^]^ The anti‐tumor ex‐srNP was initially shielded with a PEG layer during blood circulation. After irradiating tumor sites, the shells were detached and exposed to a surface with positive charges, thus facilitating the endocytosis in tumor cells and drastically enhancing the cytotoxic effect of the released drugs. Notably, non‐ionizing and non‐invasive features of the SDT repeated the stimuli and induced no toxic and side effects. The results demonstrated that the US‐responsive delivery vehicles were tempting carriers for precise spatiotemporal control of drug release, thus selectively amplified cytotoxic potency to the US‐imposed site.^[^ ^183^ ^]^

Combining immune drugs, for example, PD‐L1/PD1, with other therapies is another promising strategy for cancer treatments. Prior to that, researchers have demonstrated that combined anti‐PD‐L1 with amplified sonosensitizers of SDT induced immune cancer response, which killed tumor and prevented metastasis.^[^ ^184^ ^]^ These methods represent a combinatorial proof‐of‐concept based on non‐invasive tumor immunotherapy.^[^ ^185^ ^]^ In addition, the combination of CDT and SDT eliminated cancer cells, inhibited the expression of metastatic protein, and induced immune response by releasing TAAs. Liu et al. also developed a chondroitin sulfate/Ce6/lipoic acid nano‐platform loaded with docetaxel, CT, and SDT for anti‐proliferation and anti‐metastasis.^[^ ^186^ ^]^ The above‐mentioned studies presented convenient approaches to produce multifunctional smart ex‐srNPs for efficient tumor therapy.

### 3.3 Magnetic Field‐Mediated ex‐srNPs

The lack of selectivity toward tumor masses causes severe toxicity in the body. The applications of ex‐srNPs in cancers have been researched as promising tools for improving the specificity, security, and bioavailability of traditional agents.^[^ ^187^ ^]^ Compared with a photo‐mediated response, the magnetic field barely interacts with the body. Hence, it is considered one of the best external triggers for nanocarriers.^[^ ^188^ ^]^ Tong et al. reported magnetic Fe_3_O_4_ NP, which was effectively heated by alternating magnetic fields (AMF) in tumor tissues at a minimal side effect.^[^ ^189^ ^]^ In addition, preferential targeting cancer behavior of the ex‐srNP primarily resulted from fast blood vessel growth and inefficient lymphatic drainage of defective tumor tissue vasculature.^[^ ^190^ ^]^

Upon exposure to an AMF, magnetic‐mediated ex‐srNPs dissolved in solution and transferred the magnetic waves to heat energy, and this is referred to as the magnetic heating fluid phenomenon.^[^ ^191^ ^]^ Hyperthermia treatment is proven to be an efficient tumor therapy due to the temperature upregulation of ferromagnetic or paramagnetic materials along with the intensity change of the magnetic field.^[^ ^192^ ^]^ Optimizing hyperthermia is crucial for designing heat therapeutics ex‐srNPs for highly efficient heat induction. The materials and geometric properties are modulated to increase the heat efficiency upon a clinical AMF.^[^ ^193^ ^]^ In theory, the heat production of ex‐srNPs relies on their characters, for example, rate of magnetic relaxation and magnetizing saturation. Notably, the maghemite (γ‐Fe_2_O_3_) and Fe_2_O_3_ ex‐srNP features are attractive in magnetizing saturation and magnetic relaxation.^[^ ^194^ ^]^ The magnetic Fe_3_O_4_ material ex‐srNPs are noticed by modulating their biocompatibility surface in the applications of nanomedicine. Fe_2_O_3_ ex‐srNPs modified with a polymer, such as PEG and chitosan, prevent aggregation and prolong their blood circulation.^[^ ^195^ ^]^

**Figure 7:**
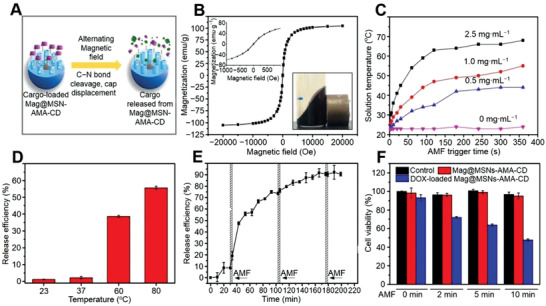
Magnetic‐mediated ex‐srNP for smart drug delivery and synergistic cancer immunotherapy. A) Schematic illustration showing formation of magnetic field inducible drug‐eluting core@shell structure nanoparticle (Mag@MSN‐AMA‐CD NP) and its application in image‐guided synergistic therapy. B) Field‐dependent magnetization curve of MnFe_2_O_4_@CoFe_2_O_4_ at 300 K. The inset shows the small scale of field‐dependent magnetization curves and how a magnet attracts the MnFe_2_O_4_@CoFe_2_O_4_@MSN@core@shell NP in hexane. C) Time‐ and concentration‐dependent temperature‐increase profile of toluene solution containing MnFe_2_O_4_@CoFe_2_O_4_ triggered by an AMF. D) Release efficiency of fluorescein from Mag@MSN after the bulk heating at 23, 37, 60, or 80 °C triggers for 10 min (*n =* 3). Mn denotes magnetic MnFe_2_O_4_@CoFe_2_O_4_, and MSN denotes mesoporous silica NP. E) Time‐dependent release profile of fluorescein from Mag@MSN through magnetic actuation under AMF for three min for 3 cycles (*n =* 3). The temperature of the solution right after each exposure is measured to be 26 °C, 3 °C higher than that before each AMF exposure. F) Viability of PANC‐1 after treatment with Mag@MSNs‐AMA‐CD or DOX‐loaded Mag@MSNs‐AMA‐CD. The control is cells without treatment by nanoparticle. The cells are treated for 4 h at a concentration of 50 µg mL^−1^ followed by 2, 5, or 10 min of AMF exposure. The cells are allowed to grow in the regular culture medium for 12 h. AMA, 1‐adamantylamine; CD, β‐cyclodextrin. Data are represented as mean ± SD (*n* = 3). Reproduced with permission.^[^ ^196^ ^]^ Copyright 2019, American Chemical Society.

The non‐invasive magnetic field‐responsive superparamagnetic ex‐srNPs provide the possibility for temporal and spatial controlled drug delivery.^[^ ^197^ ^]^ As shown in **Figure** **7**A, the Mag@MSN‐AMA‐CD NP with a thermo‐mediated gatekeeper molecule including an aliphatic azo group was modified on the core@shell ex‐srNP surface to control the drug delivery.^[^ ^196^ ^]^ After the exposure to an AMF, generating heat removed the gatekeepers and showed a controlled cargo delivery instead of the destructing construction. As shown in Figure 7B, the saturation magnetization of the MnFe_2_O_4_@CoFe_2_O_4_ loaded by Mag@MSN‐AMA‐CD NP was detected as 105 emu g^−1^, higher than that in the single Fe_3_O_4_ NP field‐dependent magnetizing curve (80 emu g^−1^). The inset of Figure 7B showed the small‐scale field‐relevant magnetization curves and a magnet attracted MnFe_2_O_4_@CoFe_2_O_4_ NP. The stability and drug loading capacity of magnetically activated ex‐srNPs had a promising prospect for facilitating synergistic tumor hyperthermia. In addition, the focal heat of cancer and ex‐srNP areas increased the efficiency of tumor thermos‐ablative treatment.^[^ ^198^ ^]^ This ex‐srNP also showed a remarkably high tissue penetration, which was necessary for drug delivery.^[^ ^199^ ^]^

The solution temperature, including ex‐srNP agents, reached 67.0 °C after 6 min AMF radiation at the different concentrations of MnFe_2_O_4_@CoFe_2_O_4_. Under AMF stimulation, the temperature‐sensitive caps release the MnFe_2_O_4_@CoFe_2_O_4_ were developed.^[^ ^196^ ^]^ As shown in Figure 7C, concentration‐ and time‐relevant increasing temperature in MnFe_2_O_4_@CoFe_2_O_4_ solution showed an abrupt improvement under AMF inducer. A designed AMF frequency directly produced the toxicity of cancer cells above 42.0 °C and caused heating elimination as the temperature was over 50.0 °C, inducing the coagulation and necrosis of cancer cells. Compared to conventional thermal therapy, MHT offered no normal tissue damnification to control therapeutic dose delivery, which acted as a potential target magnetic hyperthermia application.^[^ ^200^ ^]^

It was reported that only 3% of fluorescein was released from Mag@MSNs‐AMA‐CD as it was heated up below 37.0 °C for 10 min, exhibiting tightly blocked pores (Figure 7D).^[^ ^196^ ^]^ As shown in Figure 7E, less than 10% fluorescein slight leakage was recorded at room temperature in the first 30 min before exposure to the AMF actuation in the first cycle. The fluorescein release plateau reached a 60% equilibrium value in the first cycle, which was analogical to the fluorescein release level for 3 min. As the AMF exposure time was increased, the DOX dosage‐controlled delivery by DOX‐loaded Mag@MSNs‐AMA‐CD increased and eliminated more tumors (Figure 7F). The ex‐srNP demonstrated that an AMF remotely triggered ex‐srNP for the DOX delivery in the intracellular microenvironment.

The superparamagnetic high magnetization of ex‐srNPs generates a significant amount of heating with optimizing the application for controlled heat drug release. Espinosa et al. designed Fe_2_O_3_ NP with the ability to act as photothermal and magnetic nanoagents.^[^ ^201^ ^]^ The ex‐srNP exhibited a high traversing rate and magnetization for guiding tumor therapy via the package of irons.^[^ ^202^ ^]^ Albarqi et al. designed ex‐srNP with biocompatible nanocluster and efficient heat energy for systemic MHT.^[^ ^203^ ^]^ Upon intravenous injection, drug‐loaded ex‐srNP conjugating RGD peptides were enriched at the tumor site, caused by the cascade effect of active and magnetic targeting.^[^ ^204^ ^]^

Among many multi‐modal therapies, the combination of immunotherapy with MHT offers a new method to eliminate cancer with minimum cytotoxicity. The mild MHT activates the systemic immune response and combines with PD‐L1 siRNA to inhibit cancer.^[^ ^205^ ^]^ After AMF radiation, the tumor‐associated drugs are exposed to the generated tumor debris and collaborated with adjuvant to offer tumor vaccine‐like functions. Combining ex‐srNPs with MHT and immune drugs leads to a systemic therapeutic response to inhibit a wide range of cancer metastasis.^[^ ^206^ ^]^ Overall, synergistic immunomodulation/ferroptosis via biomimetic magnetosome has become a promising combination anti‐tumor therapy.^[^ ^207^ ^]^ Specifically, MHT will continue to explore in preclinical research of tumors and begin to be developed in clinical tumor therapy studies.

### 3.4 Radiation‐Mediated ex‐srNPs

Radiation refers to ionizing radiation, such as X‐ray, γ‐ray, and particle radiation, or electromagnetic radiation, to kill cancer cells and prevent the further spreading of cancer cells.^[^ ^208^ ^]^ However, high‐intensity and long‐time radiation induce radio‐dermatitis, which seriously affects the life quality of the patient. Therefore, it is necessary to reduce the intensity of radiation and utilize targeted drug delivery to reduce their distribution in normal tissue for minimal side effects.^[^ ^209^ ^]^ The X‐ray‐activated ex‐srNPs achieved significant progress in developing controlled drug delivery and immune‐radiotherapy for systemic tumor elimination.^[^ ^210^ ^]^ In general, the well‐designed radiation‐mediated ex‐srNPs overcome the RT limitation with low radiating doses limitations for efficient cancer therapy.

**Figure 8:**
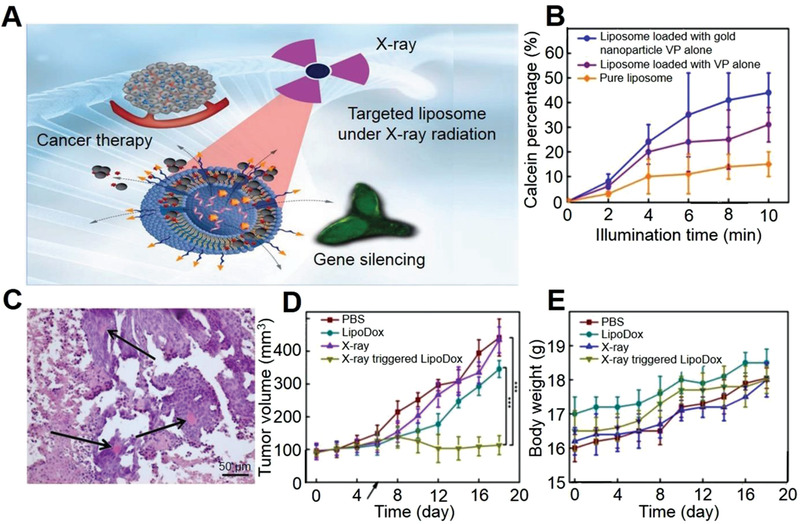
Radiation‐mediated ex‐srNP for smart drug delivery and synergistic cancer immunotherapy. A) Schematic illustration of gene silencing and cancer cells killing by X‐ray‐triggered liposome. This liposomal delivery platform incorporates VP and Au NP. Two types of cargos, antisense oligonucleotide, and DOX, are respectively entrapped inside a liposomal middle cavity to demonstrate in vitro drug delivery. B) Calcein release profiles from liposomes under 360 nm irradiation. C) Structural components of treated tumor (H&E staining). Viable tumor tissues are composed of uniform cells with basophiliccytoplasm (blue) and large roundish hyperchromatic nuclei. The areas of cellular paranecrosis and necrosis are recognized by disorganized groups of tumor cells with eosinophilic (pink) cytoplasm, with and without nuclei, respectively. Arrows indicate congested blood vessels. Note the spatial association between the viable tumor tissue and blood vessels. The scale bar is 50 µm. D) Changes in tumor volume. E) Mouse body weight after various treatments as indicated. The mean tumor volumes are analyzed using the *t*‐test (*n* = 4; *P < 0.05, **P < 0.01, ***P < 0.001). Reproduced with permission.^[^ ^211^ ^]^ Copyright 2018, Springer Nature.

As shown in **Figure** **8**A, an X‐ray‐triggered ex‐srNP incorporating sensitizer of verteporfin (VP) and gold into liposomes was designed.^[^ ^211^ ^]^ Under X‐ray irradiation, drugs were released from the cavity of liposome membrane, and VP was activated to create ^1^O_2_. X‐ray‐mediated materials containing S─S or Se─Se were also designed as drug delivery nanocarriers.^[^ ^212^ ^]^ These radiation‐mediated delivery systems achieved the controlled release of chemotherapeutic drugs in the tumor site. In addition, bionic nano‐capsules were designed by crosslinking polythymine and photoisomerized polyazobenzene with adenine‐modified ZnS NPs.^[^ ^57^ ^]^ The ZnS NP converted X‐ray into ultraviolet (UV) radiation that isomerized the azobenzene group, which allowed the controlled diffusion of the active payloads across the bilayer membrane. These strategies enhanced the effect of CT and RT with less required radiation dose and finally achieved the synergistic anti‐tumor effect with high efficiency and low toxicity.

As shown in Figure 8B, the fluorescent intensity from liposomes incorporated with VP and Au NP reached a maximum of 44% after 10 min radiation.^[^ ^211^ ^]^ Upon radiating X‐ray, calcein was a fluorescent dye for controlled drug delivery detection from the liposomes to the surrounding cancer cells by increasing calcein fluorescent intensity. With an excellent penetration depth, X‐ray radiation liposomes provided a new approach to achieving targeted tumor accumulation and triggered delivery encapsulated drugs by liposomes.^[^ ^215^ ^]^ Chen et al. once summarized the X‐ray‐mediated cancer‐target nano‐systems that eliminated even system tumors with minimal radiation doses.^[^ ^213^ ^]^ Compared with other external stimuli, the advanced X‐ray technique as activation systems exhibits deep penetration and synergistic effects in cancer sites for biomedical imaging and tumor therapy applications.

After the uptake of radiation‐mediated ex‐srNP, the liposome incorporating VP and Au NP were released by X‐ray radiation (Figure 8C).^[^ ^211^ ^]^ As a result, the tumors had a distorted contracture with obvious necrotic tumor tissue, indicating that the ex‐srNP triggered by X‐ray showed a cascade anti‐tumor activity. Thus, the experiment therapy induced the oxidation stress and intrinsic tumor hypoxia to suppress tumor growth. In addition, the production of ROS utilized its toxicity to damage DNA either via direct ionization or indirect free radicals generation.^[^ ^214^ ^]^ The X‐ray‐activated nano scintillators for inner‐bed cancer treatment could generate reactive cytotoxicity species, for example, nitrogen or reactive oxygen, via a heat transformation of X‐ray high energy to low energy photons. Heavy metals, such as tantalum, can generate photoelectrons and auger electrons by irradiating X‐ray waves.^[^ ^215^ ^]^ Hence, ex‐srNPs modified materials can act as radio‐sensitizers for realizing an irradiation response in situ.

X‐ray radiation has been widely used in applications of external‐mediated ex‐srNPs owing to their high depth penetration in solid cancers.^[^ ^216^ ^]^ As shown in Figure 8D, the capability to inhibit tumor growth was detected in a bearing xenograft model mouse.^[^ ^211^ ^]^ The results showed that tumor sizes in the PBS‐, liposome‐, and X‐ray‐treated groups were increased by 3.0‐, 2.9‐, and 3.4‐fold along with treatment time, indicating a failure therapy. In contrast, the tumor volume of X‐ray‐mediated ex‐srNP gradually diminished with 74% lower to the PBS group under the same condition. Furthermore, the treatment with X‐ray‐mediated liposome did not observe mortality and weight loss, indicating good biocompatibility of the combining therapy (Figure 8E). Regarding cancer growth, the X‐ray‐mediated ex‐srNP showed more effective control than a single therapy strategy. After loading with different therapeutic agents, the ex‐srNP system will provide a multifunctional platform to integrate chemo‐, photo‐, and radiotherapies for realizing efficient cancer immunotherapy.^[^ ^217^ ^]^

Radiation can enhance the efficiency of synergistic cancer immunotherapy upon altering the cancer phenotype, upregulating immunogenic TAAs expression, and apoptosis.^[^ ^218^ ^]^ Furthermore, the releases of TAAs, associating damage molecule including the cytokines of pro‐inflammatory, recruit and activate specific cancer immune T cells or effector cells. The relevant vaccine‐like effect converts an immunosuppressive TME to an immunogenic state, which is sensitive to immune checkpoint blockades and adjuvants.^[^ ^219^ ^]^ On this basis, the X‐ray‐mediated diselenide bond incorporating in mesoporous organosilica ex‐srNP was reported to control the delivery of PD‐L1 inhibitors and DOX and the synergistic immuno‐ and chemo‐therapies of solid cancers.^[^ ^55^ ^]^ Overall, the X‐ray‐mediated ex‐srNP shows promising potential in controlling degradation and drug delivery for synergistic chemo‐immunotherapy.

## 4 Dual‐ and Multi‐Stimuli‐Responsive Nanoparticles (d/m‐srNPs) for Controlled Drug Release and Synergistic Cancer Immunotherapy

### 4.1 d‐srNPs

As ideal drug carriers, nanoparticles need to possess high drug loading, specific drug delivery toward pathological sites without leakage, and an efficient drug release in situ. To this end, different “intelligent” srNPs delivering drugs in response to internal and external stimuli, such as pH, redox, ROS, enzyme, photon, US, magnetism, and radiation, have been widely applied.^[^ ^220^ ^]^ Especially for the unique d‐srNPs responded to a combination of two stimuli signals simultaneously or sequentially, their recognizing capability of internal or/and external signals exhibit an unprecedented accurate and efficient control over drug release, thus leading to superior in vitro and in vivo anti‐tumor efficacy.^[^ ^221^, ^222^ ^]^

d‐srNPs, including GSH/ROS, pH/photon, pH/GSH, pH/ROS, pH/US, and so forth, have been developed recently and showed a more significant impact on TME than the single‐stimuli‐responsive nanoparticles (s‐srNPs) owing to the synergistic effect of regulating methods.^[^ ^223^, ^224^ ^]^ It is worth noting that d‐srNPs generally take place at the exact location simultaneously or at different stages sequentially.^[^ ^225^ ^]^ Yang et al. reported a type of photo/ROS‐responsive srNP for realizing controlled drug release, which was loaded with photosensitizer and ROS sensitive bis‐(alkylthio) alkene linker.^[^ ^226^ ^]^ Such DOX‐loaded d‐srNP presented an on‐demand light trigger drug release and synergistic therapeutic efficiency in tumor therapy. In addition, US‐ combined with GSH‐responsive nanoparticles acted as an excellent candidate for synergistic tumor immunotherapy, owing to the ability to overcome low tissue‐penetration depth.^[^ ^227^ ^]^ To achieve a smart drug release, two different cascade stimuli are therefore rationally considered to enhance the specificity and versatility of the synergistic therapy further.^[^ ^228^ ^]^

The d‐srNPs encapsulated with drugs can sense a subtle change, including internal/external cell environment parameters.^[^ ^229^ ^]^ Our group developed a class of GSH/US dual‐responsive JNP srNPs, which were cracked into small Janus Au‐MnO*_x_* NP after US treatment.^[^ ^230^ ^]^ Furthermore, the Au‐MnO*_x_* was disassembled into small Au NP and Mn^2+^ ions in response to the high level of GSH in the cancer cells, and the production of Mn^2+^ was also acted as an MRI contrast agent for tracing tumor situation. Such smart JNP srNP integrated with CDT and SDT effect was activated by cascade dual response, exhibiting distinct production of ROS. Differences between intracellular microenvironments of cancerous and normal tissues combined with external stimuli have inspired the design of cascade d‐srNPs for controlling drug release and enhancing immunotherapy efficiency.^[^ ^231^ ^]^

These d‐srNPs delivered different damage to the tumor will minimize the side effects of anti‐tumor drugs, maximize the utilization rate of anti‐tumor drugs, and eliminate the tumor cells to the greatest extent. Firstly, neutralizing the pH value of the tumor promotes the infiltration of immune cells into the tumor. Furthermore, alleviating hypoxia and GSH depletion significantly damages TME and cellular ADS, achieving excellent cancer target therapy in vitro and in vivo. Sun et al. developed the disulfide as an oxidation‐responsive linkage for ROS‐responsive on‐demand drug release.^[^ ^232^ ^]^ Finally, the released drugs kill tumor cells for synergistic immunotherapy, and regulation of internal TME will counteract the antioxidant system of the tumor and boost the immune system to enhance the therapeutic effect.

**Figure 9:**
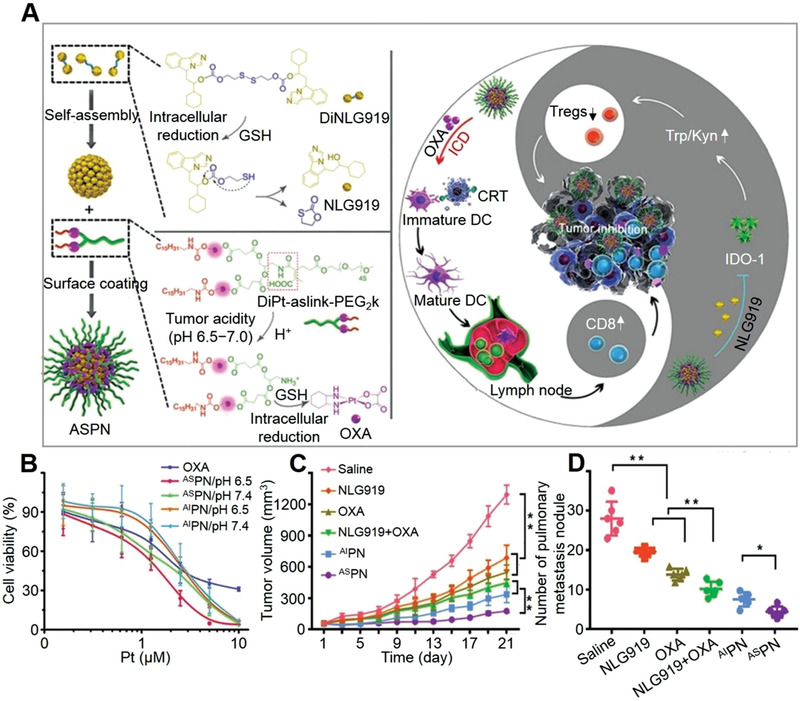
d‐srNP for triggered drug release and synergistic cancer immunotherapy. A) Schematic illustration of BCPN constructed from a tumor acidity and reduction OXA prodrug and a reduction‐activatable homodimer of NLG919 for improved immunotherapy by cooperatively modulating the immune TME. B) Cell viability of 4T1 cells examined post 48 h incubation with OXA or acid‐sensitive BCPNs (^AS^PN). Data are expressed as mean ± SD (*P < 0.05, **P < 0.01). C) Tumor growth curves in 4T1 tumor‐bearing mice following indicated treatments. Data are expressed as mean ± SD (*n* = 6; **P < 0.01). D) The number of lung metastatic nodules of mice bearing 4T1 tumors at the end of anti‐tumor study. Data are expressed as mean ± SD (*n* = 6; *P < 0.05, **P < 0.01). Reproduced with permission.^[^ ^233^ ^]^ Copyright 2018, Wiley‐VCH.

The binary cooperative efficiently activated OXA and accelerated cancer recruitment of immune T cells.^[^ ^233^ ^]^ As shown in **Figure** **9**A, the dual pH/ROS cancer‐responsive prodrug of binary cooperative prodrug nanoparticle (BCPN) was purely constructed to induce robust cancer immunity. Under acid cancer‐triggered breakage of the poly(ethylene glycol), surface charges of BCPN converted from negative to positive electrics for improving cancer deep penetration and accumulation. The cytotoxicity data suggested an increased cellular uptake and showed the highest cytotoxicity in ^AS^PN at the pH 6.5 group (Figure 9B). The NLG919 (a potent IDO‐1 inhibitor), OXA, and IDO‐1 inhibitors inducing chemo‐/immunotherapy exhibited cancer elimination efficacy (Figure 9C). Compared with the group of acid‐insensitive analogs of ^AS^PN (namely ^AI^PN), the ^AS^PN group eliminated metastatic cancer cells more efficiently. As shown in Figure 9D, the therapeutic studies demonstrated that d‐srNPs showed synergetic effect over metastasis tumor ablation in vitro and in vivo. In conclusion, these cooperative assemblies of d‐srNPs can be used as highly simplifying and efficient all‐in‐one theranostic systems for targeting imaging‐guided therapy.^[^ ^234^ ^]^

Zhang et al. reported a novel d‐srNP theranostic system with pH/GSH‐responsive drug release behavior for neutralizing pH value and activating a strong drug release.^[^ ^235^ ^]^ The thermal and pH d‐srNP simultaneously exhibited anti‐tumor and pro‐immunogenic effects as they were employed for synergistic immune‐ and microwave thermos‐therapy.^[^ ^236^ ^]^ After uptake by cancer cells, the d‐srNP exerted the multi‐function of activating cancer cell death, accompanying various tumor impairment, and realizing tumor synergistic immunotherapy. Chen et al. reported that pH and redox d‐srNP showed smart DOX release behavior upon pH 5.0 and 10.0 mM GSH conditions.^[^ ^237^ ^]^ For most tumors, d‐srNP combining with external and internal stimuli simultaneously are optimal strategies to improve the immunotherapy efficacy of patients.

### 4.2 m‐srNPs

Compared with s‐srNPs/d‐srNPs, m‐srNPs to various stimuli are more intelligent and effective in controlling drug release, making them ideal as drug delivery carriers caused by their adaptations to multiple environmental changes.^[^ ^238^ ^]^ However, the design and fabrication of the m‐srNPs may lead to synthesis difficulties and high preparation costs owing to the complex mechanism.^[^ ^239^ ^]^ It was once reported that the m‐srNP (e.g., the combinations of NIR, ROS, and GSH) improved flexibility of intracellular delivery drugs and induced preferable anti‐tumor efficacy in vivo with minimal undesired drug release at normal tissues.^[^ ^240^ ^]^

**Figure 10:**
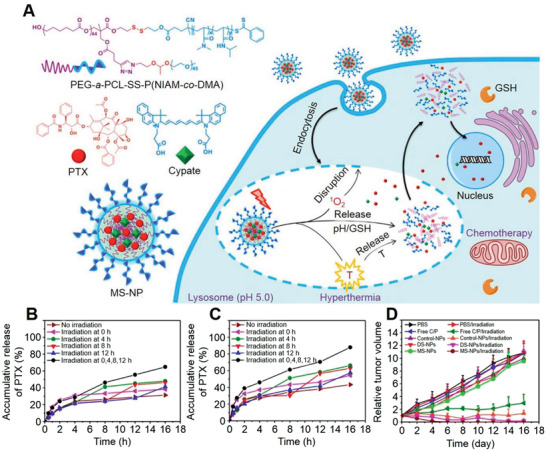
m‐srNP for triggered drug release and synergistic cancer immunotherapy. A) Schematic illustration of NIR light/pH/GSH‐responsive nanoparticle consisting of PEG‐*a*‐PCL‐SS‐P nanoparticle (NIPAM‐*co*‐DMA NP) (S1) star quaterpolymer for precise cancer therapy with synergistic effects. B) Accumulative release of PTX from m‐srNP at the concentration of 100 µg mL^−1^ Cypate at pH 7.4 under 15 min irradiation at 0, 4, 8, or 12 h. C) Accumulative release of PTX from MS‐NP at pH 5.0 under 15 min irradiation at 0, 4, 8 or 12 h. D) Tumor growth profiles of mice injected with MS‐NP, DS‐NP, normal nanoparticle (control‐NP), and free Cypate/PTX (C/P) at the dose of 7.5 mg (kg BW)^−1^ Cypate or PTX, followed by 785 nm light irradiation at 24 h post‐injection (5 min, 1.0 W cm^−2^). Reproduced with permission.^[^ ^241^ ^]^ Copyright 2016, American Chemical Society.

As shown in **Figure** **10**A, self‐assembled PEG‐*a*‐PCL‐SS‐P micelle showed NIR/pH/GSH−responsive drug release to intracellular cancer cells, which further integrated photo‐activated hyperthermia for synergistic anti‐tumor therapy and realized an efficient tumor ablation with an extremely low regrowth rate.^[^ ^241^ ^]^ The release of PTX from micelles was evaluated by irradiating at different time‐points of 0, 4, 8, and 12 h for 15 min. As shown in Figure 10B,C, the PEG‐*a*‐PCL‐SS‐P micelle loaded with drugs had a preferable release of PTX at both pH values of 5.0 and 7.4 after NIR irradiating, and multiple responsive treatments further boosted PTX release. It was revealed that both multi‐stimuli‐responsive nanoparticle (MS‐NP) and dual‐stimuli‐responsive nanoparticle (DS‐NP) had enhanced chemotherapeutic efficacy compared to PBS in the absence of irradiation (Figure 10D).

A rational design of m‐srNPs with multiple responsiveness provided a feasible strategy to realize a precisely controlled drug delivery for cancer therapy. Lei et al. successfully fabricated a pH/redox/US m‐srNP by assembling pH‐sensitive poly(2‐(diisopropylamino ethylmethacry‐late)‐*b*‐poly(ethyleneimine) diblock and GSH‐responsive gels.^[^ ^242^ ^]^ The release rate of the loaded drugs could be accelerated as they were exposed to pH/redox/US. This result demonstrated that such pH/redox/US m‐srNP had a cascade responsive effect, ensuring an efficient drug release with low drug leakage during normal blood circulation. Although the processes of self‐assembly and recognition are similar, the m‐srNPs generally exhibit a better sensitivity in controlled drug release compared to the s‐srNPs/d‐srNPs. Xiao et al. also proved that the multi‐stimuli‐responsive boronate crosslinked micelles showed superior therapeutic efficacy for ovarian cancer.^[^ ^221^ ^]^

Recently, a novel quintuple‐stimuli‐responsive nanoparticle in response to pH, temperature, light, and oxidation or reduction species has been developed.^[^ ^243^ ^]^ The micelles with uniform size were self‐assembled through quaternization reaction between bromine of *N,N′*‐bis(bromoacetyl) cystamine and nitrogen of poly(dimethylaminoethyl methacrylate). After adding a small amount of DL‐dithiothreitol, swelling at H_2_O_2_ or acidic pH, shrinking at high temperature, and UV light irradiation, such quintuple‐stimuli‐responsive nanoparticles could be de‐crosslinked. The combination of stimuli finally regulated and triggered the drug release from micelles effectively and precisely. In addition, targeting ligands, such as TF, aptamer, and antibody fragments, can also be applied onto m‐srNPs to further enhance clinical anti‐tumor efficacy through realizing a vectored drug delivery toward cancers.^[^ ^244^ ^]^ This site‐specific cancer targeting and fast drug release system would become a highly appealing aspect for future efforts because m‐srNPs significantly improved bio‐distribution and pharmacokinetics of anti‐tumor drugs, thus offering the potential therapeutic effect with decreasing side effects in vivo.^[^ ^245^ ^]^

## 5 Conclusion and Perspectives

Over the past decades, outstanding signs of progress have been achieved by combining srNPs and immune reagents for cancer immunotherapy. Many stimuli‐responsive moieties, such as amide bond, diselenium bond, and ester bond, have been introduced to different materials for assembling srNPs, which presented an excellent promotion effect on synergistic therapy through controlling drug release.^[^ ^246^ ^]^ In addition, some inorganic materials of MnO_2_, Au, Ag, and Fe_3_O_4_ have been utilized as stimuli nanocarriers.^[^ ^247^ ^]^ Combining these srNPs with CT/PDT/PTT eliminates well‐established immunosuppressive microenvironment cancer cells by regulating cancer immune sensitivity.^[^ ^248^ ^]^ Abundant researches provide a firm infrastructure for developing the srNPs‐based tumor combinational immunotherapy.^[^ ^249^ ^]^

The developed srNPs can reconstruct the TME from neutralizing pH value, alleviating hypoxia, depleting GSH, regulating ROS concentration, and regional heating in contribution to inhibit tumor defense system.^[^ ^250^ ^]^ In addition, the controlled activation of in situ PDT, PTT, immunotherapy, and chemotherapy are also shown to induce effective immune responses for enhancing immune infiltrations.^[^ ^251^ ^]^ Moreover, the released drugs will be retained at the tumor sites for improving dosage effect after blood circulation. In this context, the srNPs loaded with various immunomodulators will eventually realize the elimination of malignant tumors and establish long‐term immune memory.^[^ ^252^ ^]^ In this review, the srNPs are divided into three classifications of en‐srNPs, ex‐srNPs, and d/m‐srNPs. Meanwhile, the corresponding anti‐tumor mechanism of the srNPs for synergistic immunotherapy is listed as below: 1) srNPs are modified with different triggering materials; 2) The designed srNPs can be accumulated at the tumor site and turn “cold” tumor into “hot” tumor with increased immune sensitivity; 3) Both distant and primary tumor cells are killed by synergizing with immune drugs; 4) Long‐term memories are established to inhibit metastatic tumors and tumor recurrences. Overall, such intelligent nanocarriers combined with immune drugs provide valuable strategies and more opportunities for efficient tumor therapy in clinic.

Anticipating the future, many excellent studies are further ongoing on optimizing the combination therapy of srNPs and immunotherapy. The srNPs encapsulated with immune reagents substantially affect patients far beyond those who were treated from srNPs or immune drugs alone. This is due to the enhanced immune response and abscopal effects.^[^ ^253^ ^]^ Additionally, the srNPs synergistic immunotherapy for training immune cells can produce specific antibodies to establish immune memory to inhibit the recurrence of cancer in the cradle.^[^ ^254^ ^]^ Notably, m‐srNPs responsive to external and internal stimuli are more effective and intelligent in regulating drug release than single responsive systems.^[^ ^255^ ^]^ However, the m‐srNPs with complex mechanisms generally result in a complicated design of drug carriers, along with increased synthetic difficulties.^[^ ^224^ ^]^ In addition, accurate monitoring, quantified regulatory ability, and extreme sensitivity of the m‐srNPs toward the TME all remain significant challenges. Overall, integrating immunotherapy mechanisms and new science of multi‐azimuth smart materials boosts synergistic cancer therapy, thus lengthening patient lives. Under ongoing research work in synergistic tumor nanotechnology and immunology, we believe that the clinical translation of the srNPs will be realized soon in the future.

## Conflict of Interest

The authors declare no conflict of interest.
